# Publisher Correction: Experimental determination of partial charges with electron diffraction

**DOI:** 10.1038/s41586-025-09608-5

**Published:** 2025-09-15

**Authors:** Soheil Mahmoudi, Tim Gruene, Christian Schröder, Khalil D. Ferjaoui, Erik Fröjdh, Aldo Mozzanica, Kiyofumi Takaba, Anatoliy Volkov, Julian Maisriml, Vladimir Paunović, Jeroen A. van Bokhoven, Bernhard K. Keppler

**Affiliations:** 1 Department of Inorganic Chemistry, University of Vienna, Vienna, Austria; 2 Vienna Doctoral School in Chemistry (DoSChem), University of Vienna, Vienna, Austria; 3 Department of Computational Biological Chemistry, University of Vienna, Vienna, Austria; 4 Center for Photon Science, Paul Scherrer Institute, Villigen, Switzerland; 5 Department of Chemistry, Middle Tennessee State University, Murfreesboro, TN USA; 6 Quantum Optics, Quantum Nanophysics and Quantum Information, University of Vienna, Vienna, Austria; 7 Institute for Chemical and Bioengineering, ETH Zurich, Zurich, Switzerland; 8 PSI Center for Energy and Environmental Sciences, Paul Scherrer Institute, Villigen, Switzerland

**Keywords:** Analytical chemistry, Structural materials, Computational chemistry, Structure elucidation, Characterization and analytical techniques

Correction to: *Nature* 10.1038/s41586-025-09405-0 Published online 20 August 2025

In the version of this article initially published, in the first sentence of the Methods “Data processing and model refinement” section, reading “Data were processed with XDS,” a citation to what is now ref. 42 was missing. The reference (Kabsch, W. XDS. *Acta Crystallogr., Sect. D: Biol. Crystallogr*. **66**, 125–132 (2010)) is now included in the HTML and PDF versions of the article.

