# Supplementary material for: Experimental determination of partial charges with electron diffraction
Source: Nature. 2025 Aug 20;645(8079):88–94. doi: 10.1038/s41586-025-09405-0 (PMC12408337; doi:10.1038/s41586-025-09405-0)
Supplement: Supplementary file 1 — This file includes a glossary of (crystallographic) terms and Supplementary Tables 1–29. [file 41586_2025_9405_MOESM1_ESM.pdf]

nature portfolio

https://doi.org/10.1038/s41586-025-09405-0

Supplementary information

Experimental determination of partial charges with electron diffraction

In the format provided by the authors and unedited

Appendix S1 Supplemental Tables

S1.1 Glossary of (crystallographic) terms

R_1 The crystallographic R_1 is a measure for the difference between the observed reflection intensities and the intensities calculated from the model. For its calculation, the amplitudes, i.e. the square root of the actual intensities are used:

$$R_1 = \frac{\sum_{hkl} ||F_{\text{obs}}(hkl)| - |F_{\text{calc}}(hkl)||}{\sum_{hkl} |F_{\text{obs}}(hkl)|}$$

Abridged notation of errors Short hand for writing standard uncertainties. The figure provided in errors refers to the last digit(s) of the main figure provided, e.g.

$$0.82(5) = 0.82 \pm 0.05$$
$$0.123(12) = 0.123 \pm 0.012$$
$$167(3) = 167 \pm 3$$

S1.2 Tabulated results for Ciprofloxacin

Table S1 Experimental charges extracted from Ciprofloxacin dataset.

Atom	DS
**Cl1**	-0.50(3)
**C18**	0.11(3)
**O1**	-0.32(3)
**O3**	-0.40(3)
**C13**	-0.31(3)
**C12**	-0.18(3)
**N3**	-0.15(3)
**C7**	-0.03(3)
**C8**	-0.09(3)
**C14**	0.09(3)
**O2**	-0.14(3)
**C15**	-0.21(3)
**C17**	-0.38(3)
**C16**	-0.54(3)
**C6**	-0.39(3)
**C5**	-0.15(3)
**C10**	0.19(3)
**F1**	-0.23(3)
**C9**	-0.24(3)
**N1**	-0.34(3)
**C4**	-0.38(3)
**C3**	-0.38(3)
**N2**	-0.53(3)
**C2**	-0.38(3)
**C1**	-0.32(3)
**H1A**	0.26(3)
**H1B**	0.43(3)
**H2A**	0.30(3)
**H2B**	0.31(3)
**H2C**	0.24(3)
**H2D**	0.30(3)
**H3**	0.47(3)
**H3A**	0.34(3)
**H3B**	0.34(3)
**H4A**	0.31(3)
**H4B**	0.34(3)
**H6**	0.38(3)
**H9**	0.51(3)
**H12**	0.36(3)
**H15**	0.16(3)
**H16A**	0.21(3)
**H16B**	0.33(3)
**H17A**	0.32(3)
**H17B**	0.30(3)

Table S2 Values of U_{eq} for Ciprofloxacin, dataset DS_{mrg}, iSFAC modelling compared with conventional modelling. Note: In conventional modelling, U_{eq} is not available for hydrogen atoms, since they are refined with the riding atom model.

	$U_{eq} \times 1000$ [Å^2]	
	iSFAC	conv.
Cl(1)	32(1)	26(1)
F(1)	21(1)	20(1)
O(1)	21(1)	17(1)
N(1)	14(1)	20(1)
C(1)	16(1)	16(1)
O(2)	18(1)	11(1)
N(2)	18(1)	18(1)
C(2)	16(1)	6(1)
O(3)	19(1)	12(1)
N(3)	12(1)	22(2)
C(3)	17(1)	14(2)
C(4)	15(1)	12(2)
C(5)	13(1)	7(1)
C(6)	12(1)	7(1)
C(7)	12(1)	6(1)
C(8)	13(1)	7(1)
C(9)	14(1)	5(1)
C(10)	15(1)	7(1)
C(12)	13(1)	8(1)
C(13)	14(1)	10(1)
C(14)	15(1)	7(1)
C(15)	13(1)	8(1)
C(16)	14(1)	9(1)
C(17)	16(1)	21(2)
C(18)	17(1)	14(2)
H(1A)	18(4)	
H(1B)	19(4)	
H(2A)	27(3)	
H(2B)	20(3)	
H(2C)	19(3)	
H(2D)	17(4)	
H(3)	21(3)	
H(3A)	20(4)	
H(3B)	19(4)	
H(4A)	16(4)	
H(4B)	14(4)	
H(6)	13(4)	
H(9)	17(4)	
H(12)	15(4)	
H(15)	14(4)	
H(16A)	14(4)	
H(16B)	16(4)	
H(17A)	17(4)	
H(17B)	18(4)	

S1.3 Tabulated results for tyrosine

Table S3 Experimental charges extracted from tyrosine datasets.

Atom	DS1	DS2	DS3	DS4	DS5	DS6	DS7	DS8	DS9	DS_mrg
**C1**	-0.17(9)	0.07(8)	-0.17(6)	-0.14(9)	-0.20(5)	-0.26(7)	0.00(6)	-0.14(7)	-0.17(6)	-0.15(6)
**C2**	-0.26(9)	-0.43(8)	-0.49(8)	-0.23(9)	-0.41(7)	-0.39(7)	-0.49(7)	-0.41(8)	-0.37(7)	-0.30(7)
**H2**	0.27(6)	0.39(5)	0.37(5)	0.27(5)	0.33(4)	0.35(5)	0.41(5)	0.35(5)	0.30(5)	0.29(4)
**C3**	-0.25(9)	-0.07(8)	-0.08(8)	-0.18(8)	-0.14(6)	-0.10(7)	-0.02(7)	-0.14(8)	-0.13(6)	-0.10(6)
**H3**	0.24(5)	0.23(5)	0.24(5)	0.29(5)	0.23(4)	0.20(4)	0.28(5)	0.24(5)	0.23(4)	0.20(4)
**C4**	-0.31(8)	-0.17(7)	-0.18(7)	-0.14(8)	-0.15(6)	-0.10(6)	-0.16(7)	-0.19(8)	-0.13(6)	-0.11(6)
**O2**	-0.40(10)	-0.45(9)	-0.42(8)	-0.45(10)	-0.26(7)	-0.33(8)	-0.05(8)	-0.42(9)	-0.34(7)	-0.27(7)
**H2A**	0.26(6)	0.33(6)	0.33(5)	0.24(6)	0.30(4)	0.24(5)	0.28(5)	0.30(5)	0.29(5)	0.23(4)
**C5**	-0.35(9)	-0.35(8)	-0.34(7)	-0.36(8)	-0.21(6)	-0.26(6)	-0.28(7)	-0.32(8)	-0.25(6)	-0.25(6)
**H5**	0.41(6)	0.33(5)	0.33(5)	0.34(5)	0.34(4)	0.39(5)	0.21(4)	0.33(5)	0.35(4)	0.29(5)
**C6**	-0.18(8)	-0.12(7)	-0.19(7)	-0.15(8)	-0.26(6)	-0.29(7)	-0.36(6)	-0.14(7)	-0.23(7)	-0.28(6)
**H6**	0.28(6)	0.22(5)	0.23(5)	0.28(6)	0.33(4)	0.37(5)	0.21(4)	0.25(5)	0.31(5)	0.33(5)
**C7**	-0.26(9)	-0.46(8)	-0.40(7)	-0.36(9)	-0.36(7)	-0.26(7)	-0.38(7)	-0.42(7)	-0.35(8)	-0.38(7)
**H7A**	0.29(6)	0.36(5)	0.30(5)	0.32(5)	0.30(4)	0.34(5)	0.30(5)	0.27(5)	0.32(4)	0.29(4)
**H7B**	0.30(6)	0.23(5)	0.34(5)	0.28(5)	0.30(4)	0.31(5)	0.23(4)	0.34(5)	0.28(5)	0.32(5)
**C8**	-0.10(10)	-0.16(8)	-0.19(7)	-0.19(9)	-0.16(6)	-0.23(8)	-0.04(6)	-0.14(8)	-0.16(7)	-0.19(6)
**H8**	0.35(6)	0.34(5)	0.38(5)	0.33(6)	0.35(4)	0.37(5)	0.34(5)	0.38(6)	0.37(5)	0.30(4)
**N1**	-0.52(10)	-0.59(9)	-0.53(9)	-0.61(10)	-0.49(7)	-0.59(8)	-0.53(8)	-0.47(9)	-0.51(8)	-0.46(7)
**H1A**	0.48(6)	0.44(6)	0.50(5)	0.45(6)	0.36(4)	0.36(5)	0.47(5)	0.44(5)	0.35(5)	0.39(5)
**H1B**	0.34(6)	0.39(5)	0.40(4)	0.35(6)	0.28(4)	0.37(5)	0.28(4)	0.43(5)	0.28(5)	0.32(4)
**H1C**	0.25(6)	0.24(5)	0.20(5)	0.17(5)	0.19(4)	0.25(5)	0.12(4)	0.19(5)	0.21(4)	0.19(4)
**C9**	-0.09(10)	0.00(8)	-0.05(8)	-0.19(8)	-0.19(6)	-0.15(7)	-0.19(7)	-0.04(8)	-0.17(7)	-0.16(7)
**O1**	-0.39(10)	-0.42(8)	-0.32(7)	-0.12(9)	-0.28(6)	-0.34(7)	-0.42(7)	-0.38(8)	-0.25(7)	-0.29(6)
**O3**	-0.18(9)	-0.36(8)	-0.27(7)	-0.20(10)	-0.21(6)	-0.26(8)	-0.22(7)	-0.29(8)	-0.24(8)	-0.21(7)

Table S4 Values of U_{eq} for tyrosine, dataset DS_{mrg}, iSFAC modelling compared with conventional modelling. Note: In conventional modelling, U_{eq} is not available for hydrogen atoms, since they are refined with the riding atom model.

	$U_{eq} \times 1000$ [Å^2]	
	iSFAC	conv.
O1	40(2)	38(2)
N1	29(1)	28(2)
C1	25(1)	24(2)
O2	33(1)	30(2)
C2	27(1)	26(2)
O3	37(2)	35(2)
C3	27(1)	24(2)
C4	28(1)	24(2)
C5	26(1)	25(2)
C6	26(1)	25(2)
C7	29(1)	29(2)
C8	29(1)	27(2)
C9	32(2)	29(2)
H2A	28(8)	
H1A	28(7)	
H1B	26(8)	
H1C	25(10)	
H2	24(9)	
H3	17(10)	
H5	32(9)	
H6	25(8)	
H7A	21(8)	
H7B	36(9)	
H8	27(9)	

S1.4 Tabulated results for histidine

Table S5 Experimental charges extracted from histidine datasets.

Atom	DS1	DS2	DS3	DS4	DS5	DS6	DS7	DS8	DS9	DS_mrg
**C1**	-0.30(4)	-0.27(3)	-0.29(4)	-0.23(4)	-0.17(3)	-0.35(4)	-0.31(5)	-0.27(4)	-0.15(3)	-0.16(3)
**C2**	-0.17(4)	-0.22(4)	-0.29(4)	-0.32(4)	-0.21(3)	-0.15(4)	-0.33(6)	-0.26(4)	-0.33(3)	-0.33(3)
**H2**	0.30(3)	0.27(3)	0.37(3)	0.38(3)	0.32(2)	0.34(4)	0.31(4)	0.32(3)	0.24(2)	0.24(2)
**N1**	0.12(4)	0.13(4)	0.02(4)	0.05(4)	-0.13(3)	-0.05(4)	0.02(7)	0.01(4)	0.01(4)	0.02(4)
**H1**	0.43(3)	0.46(3)	0.30(3)	0.27(4)	0.33(3)	0.12(4)	0.43(5)	0.31(3)	0.30(3)	0.31(3)
**C3**	-0.34(4)	-0.34(3)	-0.17(4)	-0.16(4)	-0.19(3)	0.00(4)	-0.31(6)	-0.17(4)	-0.15(3)	-0.19(3)
**H3**	0.11(3)	0.11(3)	0.24(3)	0.25(3)	0.22(2)	0.37(4)	0.10(4)	0.33(3)	0.24(3)	0.24(3)
**N2**	-0.22(4)	-0.30(4)	-0.44(4)	-0.50(4)	-0.39(3)	-0.25(4)	-0.07(6)	-0.52(4)	-0.29(3)	-0.30(3)
**C4**	-0.58(4)	-0.57(4)	-0.36(4)	-0.32(4)	-0.30(3)	-0.32(4)	-0.74(6)	-0.34(4)	-0.34(4)	-0.35(3)
**H4A**	0.20(3)	0.20(3)	0.36(3)	0.38(4)	0.38(3)	0.26(4)	0.27(4)	0.32(3)	0.26(3)	0.26(3)
**H4B**	0.66(3)	0.69(4)	0.55(3)	0.53(4)	0.38(3)	0.20(3)	0.61(5)	0.48(3)	0.36(3)	0.36(3)
**C5**	-0.24(4)	-0.22(4)	-0.05(4)	-0.12(4)	-0.20(3)	-0.18(4)	-0.21(6)	-0.06(4)	-0.16(3)	-0.16(3)
**H5**	0.31(3)	0.40(4)	0.29(3)	0.35(4)	0.25(3)	0.27(3)	0.32(5)	0.33(3)	0.27(3)	0.29(3)
**N3**	-0.35(4)	-0.37(4)	-0.40(4)	-0.52(5)	-0.41(4)	-0.50(4)	-0.34(8)	-0.45(4)	-0.33(4)	-0.37(4)
**H3A**	0.42(3)	0.49(3)	0.33(3)	0.31(4)	0.25(3)	0.29(4)	0.38(5)	0.34(3)	0.26(3)	0.26(2)
**H3B**	0.27(3)	0.30(3)	0.31(3)	0.39(4)	0.29(3)	0.26(3)	0.20(4)	0.38(3)	0.28(3)	0.30(3)
**H3C**	0.24(3)	0.18(3)	0.26(3)	0.28(4)	0.40(3)	0.43(4)	0.29(5)	0.29(3)	0.29(3)	0.30(3)
**C6**	-0.35(4)	-0.49(4)	-0.49(4)	-0.52(4)	-0.17(3)	-0.35(4)	-0.28(6)	-0.47(4)	-0.22(3)	-0.25(3)
**O1**	-0.59(4)	-0.57(4)	-0.44(4)	-0.36(4)	-0.40(3)	-0.13(4)	-0.41(8)	-0.42(4)	-0.31(4)	-0.31(3)
**O2**	0.10(4)	0.13(4)	-0.07(4)	-0.16(4)	-0.24(3)	-0.27(4)	0.08(7)	-0.15(4)	-0.23(3)	-0.18(3)

Table S6 Histidine: Two conformations for the intermolecular hydrogen bond N1–H1···O2. Comparison of partial charges and of bonding geometry.

L-HIS	D–H···A	D–H (Å)	H···A (Å)	D···A (Å)	D–H···A (°)	RE (kcal/mol)
**DS1**	N1–H1···O2	1.12(5)	1.73(5)	2.78(1)	155(4)	128
**DS5**	N1–H1···O2	1.01(4)	1.78(4)	2.79(1)	175(4)	69

Table S7 Values of U_{eq} for histidine, dataset DS_{mrg}, iSFAC modelling compared with conventional modelling. Note: In conventional modelling, U_{eq} is not available for hydrogen atoms, since they are refined with the riding atom model.

	$U_{eq} \times 1000$ [Å^2]	
	iSFAC	conv.
O1	33(1)	32(2)
N1	31(1)	25(1)
C1	24(1)	21(2)
O2	37(2)	37(2)
N2	24(1)	23(1)
C2	25(2)	27(2)
N3	21(1)	24(1)
C3	28(2)	26(2)
C4	26(2)	25(2)
C5	23(1)	19(2)
C6	28(2)	27(2)
H1	36(9)	
H2	31(9)	
H3A	5(7)	
H3B	15(7)	
H3C	13(6)	
H3	24(9)	
H4A	28(9)	
H4B	28(8)	
H5	22(8)	

S1.5 Tabulated results for zeolite ZSM-5

Table S8 Experimental charges extracted from zeolite ZSM5 with standard uncertainties in abridged notation.

**Atom**	**DS1**	**DS2**	**DS3**	**DS4**	**DS5**
T1	0.82(6)	0.75(6)	0.73(6)	0.84(6)	0.72(5)
T2	0.93(6)	0.89(6)	0.95(7)	0.96(7)	0.89(6)
T3	0.90(6)	0.92(6)	0.80(7)	1.15(7)	1.33(6)
T4	1.01(6)	0.87(6)	0.82(7)	0.93(7)	0.99(6)
T5	0.93(5)	0.93(5)	0.93(6)	0.84(6)	0.74(5)
T6	0.82(6)	0.74(6)	0.72(7)	0.76(7)	0.64(6)
T7	0.81(5)	0.81(5)	0.78(6)	0.82(6)	0.72(5)
T8	0.92(5)	0.94(6)	0.88(6)	0.82(6)	0.62(5)
T9	0.80(5)	0.58(5)	0.77(6)	0.90(6)	1.02(5)
T10	0.77(5)	0.63(5)	0.90(6)	0.70(6)	0.74(5)
T11	0.79(5)	0.97(5)	0.73(6)	0.57(6)	0.68(5)
T12	1.00(6)	0.96(6)	0.88(6)	0.81(6)	0.66(5)
O1	-0.14(5)	-0.12(5)	-0.35(6)	-0.31(5)	-0.42(4)
O2	-0.47(5)	-0.47(4)	-0.38(5)	-0.37(5)	-0.27(4)
O3	-0.29(4)	-0.34(4)	-0.37(5)	-0.22(5)	-0.34(4)
O4	-0.51(4)	-0.42(4)	-0.46(5)	-0.40(5)	-0.37(4)
O5	-0.21(6)	-0.33(5)	-0.16(7)	-0.31(6)	-0.25(5)
O6	-0.53(5)	-0.59(5)	-0.39(7)	-0.98(6)	-0.92(5)
O7	-0.32(5)	-0.42(5)	-0.34(6)	-0.24(6)	-0.44(5)
O8	-0.29(5)	-0.45(5)	-0.10(6)	-0.22(6)	-0.08(5)
O9	-0.43(6)	-0.45(5)	-0.55(7)	-0.31(7)	-0.38(6)
O10	-0.27(5)	-0.32(5)	-0.31(6)	-0.21(6)	-0.19(5)
O11	-0.47(5)	-0.40(4)	-0.38(6)	-0.42(6)	-0.36(5)
O12	-0.50(5)	-0.43(4)	-0.34(6)	-0.55(6)	-0.40(5)
O13	-0.26(5)	-0.36(4)	-0.39(5)	-0.18(5)	-0.32(4)
O14	-0.54(5)	-0.54(4)	-0.42(6)	-0.54(6)	-0.53(5)
O15	-0.23(5)	-0.11(5)	-0.30(6)	-0.16(6)	-0.11(5)
O16	-0.44(4)	-0.23(5)	-0.48(5)	-0.36(5)	-0.48(4)
O17	-0.66(4)	-0.48(4)	-0.56(5)	-0.45(5)	-0.34(4)
O18	-0.35(6)	-0.43(6)	-0.36(7)	-0.60(7)	-0.49(6)
O19	-0.45(5)	-0.59(4)	-0.30(6)	-0.41(6)	-0.34(5)
O20	-0.56(5)	-0.56(4)	-0.62(5)	-0.34(5)	-0.54(4)
O21	-0.34(6)	-0.10(6)	-0.05(8)	-0.34(8)	-0.23(7)
O22	-0.30(5)	-0.31(4)	-0.39(6)	-0.26(6)	-0.27(5)
O23	-0.51(6)	-0.18(6)	-0.44(7)	-0.69(8)	-0.43(6)
O24	-0.61(5)	-0.60(4)	-0.63(5)	-0.50(5)	-0.54(4)
O25	-0.47(4)	-0.41(4)	-0.53(5)	-0.45(5)	-0.39(4)
O26	-0.33(6)	-0.35(6)	-0.31(8)	-0.29(8)	-0.32(7)
$\sum(T)$	10.50	9.99	9.89	10.10	9.75
$\sum(O)$	-10.48	-9.99	-9.91	-10.11	-9.75
$\sum(T+O)$	0.02	0.00	-0.02	-0.01	0.00

Table S9 Values of U_{eq} for ZSM-5, dataset DS3, iSFAC modelling compared with conventional modelling.

	$U_{eq} \times$ 1000 [Å^2]	
	iSFAC	conv.
T(1)	23(1)	21(1)
T(2)	33(1)	29(1)
T(3)	28(1)	26(1)
T(4)	25(1)	23(1)
T(5)	22(1)	20(1)
T(6)	28(1)	26(1)
T(7)	28(1)	25(1)
T(8)	32(1)	29(1)
T(9)	26(1)	24(1)
T(10)	26(1)	24(1)
T(11)	26(1)	24(1)
T(12)	28(1)	26(1)
O(1)	25(2)	36(1)
O(2)	35(2)	49(2)
O(3)	28(2)	42(2)
O(4)	28(2)	44(2)
O(5)	32(2)	40(2)
O(6)	39(2)	57(2)
O(7)	36(2)	52(2)
O(8)	34(2)	43(2)
O(9)	31(2)	49(2)
O(10)	33(2)	46(2)
O(11)	33(2)	47(2)
O(12)	28(2)	41(2)
O(13)	29(2)	40(2)
O(14)	27(2)	43(2)
O(15)	30(2)	40(2)
O(16)	31(2)	44(2)
O(17)	34(2)	51(2)
O(18)	27(2)	39(2)
O(19)	33(2)	44(2)
O(20)	36(2)	54(2)
O(21)	28(2)	34(2)
O(22)	28(1)	41(1)
O(23)	24(2)	35(2)
O(24)	33(2)	54(2)
O(25)	28(2)	40(2)
O(26)	29(2)	39(2)

Table S10 Partial charges for zeolite ZSM-5 for a data set collected at $T = -110°C$ and a data set collected at room temperature, $T = 21°C$. FVAR: value of respective free variable in SHELXL refinement; esu: estimated standard uncertainty of FVAR; q: resulting partial charge, for T-sites: $q = 4×FVAR$, for O: $q = -FVAR$; Ueq: equivalent ADP value.

	$T = -110°C$				$T = 21°C$			
ATOM	FVAR	esu	q[e]	Ueq	FVAR	esu	q[e]	Ueq
T1	0.240	0.02	0.960	0.033	0.199	0.02	0.79	0.036
T2	0.206	0.02	0.825	0.038	0.186	0.02	0.74	0.041
T3	0.208	0.02	0.834	0.037	0.197	0.02	0.79	0.038
T4	0.189	0.02	0.758	0.030	0.227	0.02	0.91	0.035
T5	0.268	0.02	1.073	0.028	0.322	0.02	1.29	0.034
T6	0.195	0.02	0.780	0.039	0.222	0.02	0.89	0.041
T7	0.227	0.02	0.907	0.032	0.263	0.02	1.05	0.035
T8	0.188	0.02	0.754	0.034	0.215	0.02	0.86	0.038
T9	0.225	0.02	0.902	0.031	0.238	0.02	0.95	0.036
T10	0.187	0.02	0.749	0.034	0.203	0.02	0.81	0.037
T11	0.236	0.02	0.946	0.033	0.240	0.02	0.96	0.038
T12	0.246	0.02	0.986	0.037	0.265	0.02	1.06	0.039
O1	0.200	0.08	-0.200	0.040	0.203	0.09	-0.20	0.046
O2	0.231	0.08	-0.231	0.050	0.411	0.09	-0.41	0.052
O3	0.317	0.07	-0.317	0.044	0.329	0.07	-0.33	0.049
O4	0.570	0.07	-0.570	0.047	0.632	0.07	-0.63	0.049
O5	0.201	0.09	-0.201	0.047	0.344	0.09	-0.34	0.046
O6	0.827	0.09	-0.827	0.054	0.905	0.09	-0.91	0.054
O7	0.549	0.08	-0.549	0.052	0.752	0.08	-0.75	0.051
O8	0.535	0.08	-0.535	0.049	0.498	0.09	-0.50	0.051
O9	0.365	0.08	-0.365	0.049	0.365	0.09	-0.37	0.051
O10	0.435	0.08	-0.435	0.049	0.467	0.08	-0.47	0.051
O11	0.407	0.07	-0.407	0.043	0.422	0.08	-0.42	0.048
O12	0.390	0.07	-0.390	0.043	0.540	0.08	-0.54	0.046
O13	0.290	0.08	-0.290	0.048	0.289	0.08	-0.29	0.047
O14	0.360	0.07	-0.360	0.044	0.401	0.08	-0.40	0.044
O15	0.341	0.07	-0.341	0.045	0.225	0.08	-0.23	0.046
O16	0.404	0.07	-0.404	0.042	0.628	0.08	-0.63	0.042
O17	0.313	0.07	-0.313	0.046	0.403	0.07	-0.40	0.046
O18	0.406	0.09	-0.406	0.038	0.443	0.10	-0.44	0.043
O19	0.269	0.07	-0.269	0.044	0.465	0.08	-0.47	0.047
O20	0.626	0.07	-0.626	0.049	0.663	0.08	-0.66	0.053
O21	0.421	0.10	-0.421	0.037	0.146	0.11	-0.15	0.042
O22	0.158	0.07	-0.158	0.042	0.118	0.08	-0.12	0.047
O23	0.360	0.10	-0.360	0.040	0.247	0.10	-0.25	0.043
O24	0.643	0.07	-0.643	0.052	0.679	0.07	-0.68	0.054
O25	0.418	0.07	-0.418	0.047	0.421	0.08	-0.42	0.049
O26	0.436	0.11	-0.436	0.041	0.108	0.12	-0.11	0.043
sum			-3.60E-04			-3.70E-04		

S1.6 Parameterisation of electron scattering factors of ions and atoms

Table S11 Cromer-Mann parameters and R_{scat} [50] of ionic electron scattering factors. $R_{\mathrm{scat}} = \frac{\sum_s |f_{\mathrm{MB}}(s) - f_{\mathrm{CM}}(s)|}{\sum_s |f_{\mathrm{MB}}(s)|}$. R_{scat} is accumulative, and does not differentiate between systematic and random deviations between f_{MB} and f_{CM}. The much lower values of the last column, $R_{\mathrm{nl}} = \frac{|\sum_s f_{\mathrm{MB}}(s) - f_{\mathrm{CM}}(s)|}{\sum_s |f_{\mathrm{MB}}(s)|}$ indicates that the deviations are mainly due to fluctuations above and below. Note that the latter is given as ‰, while the former is listed as %. f_{MB} is the atomic scattering factor computed with the Mott-Bethe formula via X-ray scattering factors according to Eq. 2, f_{CM} refers to the scattering factors computed with the Cromer-Mann parametrization with Eq. 1.

**Parameters:**	a_1	a_2	a_3	a_4	b_1	b_2	b_3	b_4	c	R_{scat} [%]	R_{nl} [‰]
Ciprofloxacin											
**Res. [Å]**					0.7-19						
**Ion**											
Cl^-	-64.844	2179.337	-19.211	643.065	-5.892	168.388	2.67	8.614	0.498	0.121	0.030
F^-	-62.584	1881.133	-16.193	464.448	-3.942	95.754	0.901	3.057	0.082	0.146	0.001
O^-	-64.675	2095.695	-5.325	133.097	-71.572	-0.021	-18.479	579.274	72.451	0.309	0.100
N^-	-65.549	2231.039	-19.891	663.164	-83.515	-0.02	-6.333	160.786	84.385	0.426	0.314
C^-	-68.53	2526.503	-7.849	200.334	-90.191	-0.019	-23.445	806.628	91.023	0.606	0.328
C^+	62.099	1820.496	4.068	75.137	15.609	427.287	2.29	10.163	0.302	0.184	0.019
H^+	63.227	1922.675	16.614	482.652	4.233	100.457	1.177	13.367	0.129	0.152	0.012
Tyrosine and histidine											
**Res. [Å]**					0.75-19						
**Ion**											
O^-	-64.367	2062.048	-5.091	128.016	-65.623	-0.025	-18.101	560.917	66.524	0.277	0.221
N^-	-66.498	2326.457	-8.564	183.175	-82.318	-0.028	-21.237	724.996	83.209	0.380	0.146
N^+	62.403	1876.169	4.124	89.527	16.225	460.188	2.321	10.804	0.415	0.172	0.075
C^-	-66.299	2342.395	-8.564	183.175	-82.318	-0.028	-21.237	724.996	83.209	0.555	0.837
**Res. [Å]**					0.85-16						
Zeolite ZSM-5											
**Ion**											
Si^{4+}	151.938	1242.727	44.099	340.701	14.035	76.305	5.774	13.808	0.954	0.059	0.0001
O^-	-63.675	-4.530	-81.469	-17.258	1984.911	115.353	-0.026	517.953	82.427	0.147	0.012

S1.7 DFT calculation tables

S1.7.1 Ciprofloxacin

Table S12 Ciprofloxacin: B3LYP/6-31g.

Atom	AtomQM	Mulliken	Loewdin	Hirshfeld	ADCH	CM5	NPA	AIM	Becke	MK	CHELPG	RESP
**Cl1**	Cl1	-0.40	-0.40	-0.35	-0.43	-0.39	-0.84	-0.55	-0.43	-0.41	-0.42	-0.41
**C18**	C36	0.60	0.19	0.19	0.25	0.25	0.81	1.68	0.55	0.72	0.71	0.71
**O1**	O4	-0.50	-0.29	-0.30	-0.36	-0.35	-0.61	-1.21	-0.57	-0.51	-0.52	-0.51
**O3**	O5	-0.52	-0.23	-0.20	-0.29	-0.38	-0.66	-1.18	-0.55	-0.58	-0.58	-0.58
**H3**	H6	0.35	0.23	0.13	0.25	0.32	0.48	0.64	0.38	0.43	0.43	0.43
**C13**	C33	-0.12	-0.14	-0.06	-0.08	-0.05	-0.31	-0.03	-0.09	-0.42	-0.37	-0.38
**C12**	C34	0.12	0.01	0.05	-0.02	0.07	0.10	0.49	0.09	0.04	0.10	0.02
**H12**	H35	0.17	0.14	0.06	0.14	0.14	0.27	0.10	0.25	0.10	0.08	0.11
**N3**	N11	-0.57	0.00	0.01	0.01	-0.25	-0.36	-1.26	-0.41	0.01	-0.10	0.04
**C7**	C29	0.31	0.06	0.05	0.04	0.11	0.19	0.39	0.38	0.08	0.15	0.04
**C8**	C28	0.03	-0.06	-0.03	-0.05	-0.02	-0.15	0.00	0.03	-0.11	-0.12	-0.09
**C14**	C32	0.41	0.14	0.12	0.16	0.15	0.49	0.94	0.43	0.54	0.51	0.51
**O2**	O3	-0.59	-0.32	-0.26	-0.33	-0.33	-0.64	-1.18	-0.57	-0.55	-0.55	-0.54
**C15**	C37	0.03	-0.04	0.02	-0.08	0.01	-0.09	0.32	0.11	0.15	0.17	0.19
**H15**	H38	0.14	0.11	0.05	0.12	0.11	0.26	0.02	0.06	0.05	0.04	0.04
**C17**	C39	-0.24	-0.20	-0.06	-0.20	-0.16	-0.46	0.03	-0.17	-0.48	-0.34	-0.54
**H17B**	H40	0.14	0.13	0.05	0.14	0.11	0.26	0.02	0.08	0.19	0.14	0.21
**H17A**	H41	0.15	0.13	0.06	0.13	0.11	0.26	0.03	0.12	0.21	0.17	0.21
**C16**	C42	-0.22	-0.21	-0.06	-0.22	-0.17	-0.49	0.02	-0.19	-0.08	-0.09	-0.01
**H16B**	H43	0.15	0.13	0.05	0.13	0.11	0.27	0.03	0.12	0.09	0.08	0.06
**H16A**	H44	0.13	0.12	0.05	0.13	0.10	0.25	0.01	0.07	0.09	0.08	0.06
**C6**	C30	-0.17	-0.18	-0.08	0.02	-0.12	-0.31	-0.01	-0.30	-0.30	-0.28	-0.27
**H6**	H31	0.10	0.12	0.04	-0.01	0.10	0.24	0.01	0.12	0.12	0.11	0.11
**C5**	C24	0.26	0.04	0.04	-0.01	0.10	0.14	0.43	0.24	0.21	0.22	0.19
**C10**	C25	0.33	0.12	0.09	0.17	0.10	0.37	0.49	0.03	0.17	0.13	0.17
**F1**	F2	-0.29	-0.13	-0.09	-0.13	-0.12	-0.34	-0.64	-0.14	-0.15	-0.15	-0.15
**C9**	C26	-0.18	-0.10	-0.04	-0.14	-0.08	-0.22	0.05	-0.31	-0.19	-0.15	-0.20
**H9**	H27	0.14	0.15	0.06	0.14	0.12	0.29	0.08	0.23	0.18	0.16	0.18
**N1**	N7	-0.49	-0.12	-0.07	-0.12	-0.32	-0.46	-1.09	-0.25	-0.26	-0.29	-0.25
**C4**	C12	-0.06	-0.12	0.00	-0.11	-0.05	-0.29	0.40	0.00	-0.13	-0.05	-0.14
**H4B**	H13	0.12	0.11	0.04	0.09	0.10	0.25	-0.01	0.06	0.09	0.05	0.10
**H4A**	H14	0.13	0.12	0.05	0.11	0.11	0.27	0.01	0.08	0.12	0.08	0.10
**C3**	C15	-0.07	-0.13	0.00	-0.12	-0.05	-0.28	0.36	-0.01	-0.03	0.10	-0.04
**H3A**	H16	0.14	0.12	0.04	0.09	0.11	0.27	0.01	0.10	0.04	0.00	0.06
**H3B**	H17	0.13	0.12	0.05	0.12	0.11	0.28	0.01	0.06	0.06	0.01	0.06
**N2**	N8	-0.53	-0.20	-0.05	-0.14	-0.46	-0.64	-1.02	-0.23	0.26	0.23	0.24
**H2A**	H9	0.21	0.16	0.06	0.14	0.16	0.46	0.39	0.09	-0.06	-0.04	-0.05
**H2B**	H10	0.27	0.19	0.13	0.22	0.31	0.44	0.38	0.25	0.12	0.09	0.13
**C2**	C18	-0.06	-0.13	0.00	-0.12	-0.05	-0.29	0.36	-0.01	-0.05	0.07	-0.21
**H2C**	H19	0.13	0.12	0.05	0.13	0.11	0.27	0.01	0.06	0.05	0.01	0.12
**H2D**	H20	0.15	0.12	0.04	0.10	0.11	0.27	0.02	0.12	0.09	0.03	0.12
**C1**	C21	-0.08	-0.12	0.00	-0.11	-0.05	-0.29	0.39	-0.02	-0.10	-0.05	0.02
**H1B**	H22	0.15	0.13	0.04	0.11	0.11	0.28	0.05	0.12	0.12	0.10	0.07
**H1A**	H23	0.11	0.10	0.03	0.09	0.10	0.24	-0.01	0.05	0.08	0.05	0.07

Table S13 Ciprofloxacin: B3LYP/6-311g.

Atom	AtomQM	Mulliken	Loewdin	Hirshfeld	ADCH	CM5	NPA	AIM	Becke	MK	CHELPG	RESP
**Cl1**	Cl1	-0.61	-0.47	-0.49	-0.50	-0.51	-0.86	-0.52	-0.68	-0.60	-0.60	-0.60
**C18**	C36	0.46	-0.09	0.19	0.27	0.25	0.80	1.56	0.58	0.80	0.76	0.79
**O1**	O4	-0.35	-0.10	-0.29	-0.37	-0.34	-0.60	-1.11	-0.58	-0.53	-0.53	-0.53
**O3**	O5	-0.32	0.08	-0.19	-0.29	-0.37	-0.63	-1.12	-0.56	-0.61	-0.60	-0.61
**H3**	H6	0.26	0.00	0.12	0.25	0.32	0.46	0.65	0.39	0.45	0.44	0.44
**C13**	C33	-0.48	-0.15	-0.06	-0.08	-0.05	-0.30	-0.03	-0.12	-0.51	-0.40	-0.47
**C12**	C34	0.26	0.08	0.05	-0.02	0.07	0.13	0.42	0.12	0.07	0.12	0.04
**H12**	H35	0.15	0.02	0.06	0.14	0.13	0.23	0.13	0.25	0.11	0.08	0.11
**N3**	N11	-0.48	0.16	0.01	0.04	-0.24	-0.38	-1.19	-0.41	0.01	-0.10	0.05
**C7**	C29	0.32	0.01	0.05	0.04	0.11	0.20	0.37	0.46	0.12	0.15	0.08
**C8**	C28	-0.24	-0.07	-0.03	-0.05	-0.02	-0.15	-0.01	0.03	-0.17	-0.12	-0.13
**C14**	C32	0.45	-0.03	0.11	0.17	0.15	0.49	0.87	0.44	0.62	0.53	0.58
**O2**	O3	-0.42	-0.12	-0.25	-0.33	-0.32	-0.65	-1.11	-0.59	-0.57	-0.57	-0.56
**C15**	C37	-0.08	0.01	0.02	-0.08	0.01	-0.04	0.28	0.10	0.14	0.17	0.17
**H15**	H38	0.15	0.01	0.05	0.12	0.11	0.21	0.04	0.05	0.06	0.04	0.04
**C17**	C39	-0.24	0.02	-0.05	-0.19	-0.16	-0.38	-0.04	-0.16	-0.50	-0.34	-0.57
**H17B**	H40	0.14	0.02	0.05	0.14	0.11	0.22	0.06	0.08	0.20	0.14	0.22
**H17A**	H41	0.16	0.02	0.05	0.13	0.11	0.22	0.06	0.11	0.22	0.16	0.22
**C16**	C42	-0.20	0.01	-0.06	-0.21	-0.16	-0.41	-0.05	-0.18	-0.08	-0.07	0.02
**H16B**	H43	0.16	0.02	0.05	0.12	0.11	0.23	0.07	0.12	0.09	0.06	0.06
**H16A**	H44	0.14	0.01	0.05	0.13	0.10	0.21	0.05	0.06	0.09	0.08	0.06
**C6**	C30	-0.10	-0.05	-0.07	-0.02	-0.12	-0.29	-0.03	-0.53	-0.34	-0.29	-0.31
**H6**	H31	0.13	0.00	0.03	0.02	0.10	0.22	0.05	0.22	0.14	0.11	0.13
**C5**	C24	0.12	-0.03	0.04	0.00	0.10	0.15	0.41	0.35	0.19	0.20	0.17
**C10**	C25	0.27	-0.01	0.08	0.22	0.10	0.38	0.45	-0.11	0.20	0.16	0.21
**F1**	F2	-0.24	0.01	-0.09	-0.16	-0.11	-0.36	-0.63	-0.10	-0.18	-0.18	-0.18
**C9**	C26	-0.05	0.02	-0.03	-0.17	-0.08	-0.18	0.04	-0.30	-0.19	-0.14	-0.20
**H9**	H27	0.13	0.03	0.06	0.15	0.12	0.25	0.12	0.25	0.19	0.17	0.19
**N1**	N7	-0.43	0.05	-0.06	-0.13	-0.32	-0.47	-1.04	-0.25	-0.22	-0.25	-0.21
**C4**	C12	-0.12	0.02	0.00	-0.10	-0.04	-0.19	0.34	0.00	-0.15	-0.06	-0.15
**H4B**	H13	0.14	0.01	0.04	0.10	0.10	0.20	0.02	0.06	0.09	0.05	0.10
**H4A**	H14	0.15	0.02	0.05	0.12	0.12	0.22	0.04	0.08	0.13	0.08	0.10
**C3**	C15	-0.09	0.04	0.01	-0.11	-0.05	-0.18	0.29	-0.02	-0.01	0.12	-0.05
**H3A**	H16	0.16	0.02	0.04	0.09	0.11	0.23	0.05	0.11	0.05	0.01	0.08
**H3B**	H17	0.15	0.02	0.05	0.13	0.12	0.23	0.04	0.07	0.06	0.01	0.08
**N2**	N8	-0.38	0.24	0.00	0.04	-0.46	-0.59	-0.99	-0.22	0.30	0.26	0.27
**H2A**	H9	0.27	-0.04	0.06	-0.05	0.20	0.47	0.46	0.28	-0.02	0.01	-0.01
**H2B**	H10	0.24	0.05	0.14	0.23	0.33	0.40	0.39	0.24	0.14	0.11	0.15
**C2**	C18	-0.10	0.04	0.01	-0.11	-0.04	-0.18	0.27	-0.02	-0.06	0.09	-0.23
**H2C**	H19	0.16	0.02	0.06	0.13	0.12	0.22	0.05	0.06	0.07	0.02	0.14
**H2D**	H20	0.16	0.02	0.05	0.10	0.12	0.22	0.06	0.13	0.10	0.03	0.14
**C1**	C21	-0.10	0.03	0.00	-0.11	-0.05	-0.19	0.33	-0.01	-0.13	-0.07	0.00
**H1B**	H22	0.16	0.02	0.05	0.12	0.12	0.24	0.09	0.12	0.14	0.11	0.08
**H1A**	H23	0.13	0.01	0.04	0.09	0.10	0.19	0.02	0.05	0.08	0.05	0.08

Table S14 Ciprofloxacin: B3LYP/def2tzvp.

Atom	AtomQM	Mulliken	Loewdin	Hirshfeld	ADCH	CM5	NPA	AIM	Becke	MK	CHELPG	RESP
**Cl1**	Cl1	-0.53	-0.35	-0.40	-0.43	-0.43	-0.85	-0.65	-0.63	-0.44	-0.44	-0.44
**C18**	C36	0.32	-0.49	0.19	0.33	0.25	0.75	1.63	0.14	0.78	0.77	0.76
**O1**	O4	-0.34	0.09	-0.30	-0.40	-0.35	-0.58	-1.19	-0.50	-0.55	-0.56	-0.54
**O3**	O5	-0.35	0.13	-0.18	-0.36	-0.37	-0.62	-1.15	-0.25	-0.59	-0.59	-0.59
**H3**	H6	0.34	0.18	0.12	0.29	0.31	0.47	0.63	0.25	0.43	0.43	0.43
**C13**	C33	-0.19	-0.13	-0.06	-0.07	-0.05	-0.32	-0.04	-0.06	-0.43	-0.38	-0.39
**C12**	C34	-0.03	-0.08	0.05	-0.06	0.08	0.13	0.45	0.19	0.03	0.11	0.01
**H12**	H35	0.19	0.17	0.06	0.13	0.13	0.24	0.12	0.32	0.11	0.08	0.11
**N3**	N11	0.05	0.29	0.01	0.07	-0.24	-0.31	-1.18	-0.32	0.04	-0.09	0.06
**C7**	C29	-0.03	-0.11	0.05	0.02	0.11	0.18	0.36	0.41	0.09	0.16	0.05
**C8**	C28	0.10	-0.08	-0.03	-0.06	-0.02	-0.16	-0.01	0.21	-0.11	-0.12	-0.08
**C14**	C32	0.20	-0.28	0.11	0.28	0.15	0.47	0.92	0.09	0.55	0.52	0.52
**O2**	O3	-0.45	0.05	-0.26	-0.41	-0.33	-0.62	-1.16	-0.40	-0.57	-0.58	-0.57
**C15**	C37	-0.12	-0.22	0.02	-0.08	0.02	-0.08	0.29	0.02	0.18	0.18	0.22
**H15**	H38	0.12	0.15	0.05	0.11	0.11	0.21	0.05	0.05	0.04	0.03	0.02
**C17**	C39	-0.21	-0.20	-0.05	-0.19	-0.16	-0.40	-0.03	-0.16	-0.49	-0.32	-0.57
**H17B**	H40	0.13	0.14	0.05	0.13	0.11	0.23	0.05	0.07	0.19	0.13	0.21
**H17A**	H41	0.15	0.14	0.05	0.13	0.11	0.23	0.06	0.11	0.21	0.16	0.21
**C16**	C42	-0.21	-0.20	-0.06	-0.21	-0.16	-0.42	-0.04	-0.17	-0.06	-0.08	0.04
**H16B**	H43	0.14	0.14	0.05	0.12	0.11	0.24	0.06	0.12	0.08	0.06	0.05
**H16A**	H44	0.13	0.13	0.05	0.13	0.10	0.22	0.05	0.06	0.09	0.08	0.05
**C6**	C30	-0.20	-0.10	-0.07	-0.07	-0.11	-0.29	-0.03	-0.49	-0.33	-0.32	-0.31
**H6**	H31	0.14	0.15	0.03	0.04	0.10	0.22	0.05	0.23	0.14	0.13	0.13
**C5**	C24	0.02	-0.17	0.04	0.01	0.10	0.14	0.42	0.36	0.18	0.22	0.16
**C10**	C25	0.37	-0.23	0.08	0.22	0.10	0.33	0.50	-0.23	0.19	0.13	0.19
**F1**	F2	-0.21	0.19	-0.09	-0.15	-0.12	-0.32	-0.66	-0.06	-0.19	-0.19	-0.19
**C9**	C26	-0.32	-0.05	-0.03	-0.20	-0.08	-0.18	0.03	-0.25	-0.19	-0.12	-0.19
**H9**	H27	0.15	0.17	0.06	0.16	0.12	0.26	0.11	0.23	0.19	0.16	0.19
**N1**	N7	-0.08	0.19	-0.06	-0.10	-0.32	-0.38	-1.06	-0.23	-0.19	-0.23	-0.18
**C4**	C12	-0.16	-0.26	0.00	-0.11	-0.05	-0.23	0.36	-0.02	-0.09	-0.01	-0.10
**H4B**	H13	0.12	0.14	0.04	0.09	0.10	0.20	0.01	0.05	0.06	0.03	0.08
**H4A**	H14	0.14	0.14	0.05	0.11	0.11	0.23	0.04	0.08	0.11	0.06	0.08
**C3**	C15	-0.19	-0.28	0.00	-0.12	-0.05	-0.23	0.30	-0.01	-0.04	0.07	-0.07
**H3A**	H16	0.15	0.14	0.04	0.09	0.11	0.23	0.04	0.10	0.04	0.01	0.07
**H3B**	H17	0.14	0.14	0.05	0.12	0.12	0.23	0.04	0.06	0.06	0.02	0.07
**N2**	N8	-0.27	0.08	-0.02	-0.08	-0.46	-0.53	-0.99	-0.35	0.35	0.31	0.32
**H2A**	H9	0.32	0.08	0.06	0.07	0.18	0.47	0.45	0.40	-0.11	-0.09	-0.10
**H2B**	H10	0.22	0.18	0.13	0.22	0.32	0.40	0.37	0.24	0.11	0.09	0.12
**C2**	C18	-0.15	-0.27	0.01	-0.11	-0.05	-0.23	0.30	-0.02	-0.09	0.04	-0.27
**H2C**	H19	0.14	0.14	0.05	0.13	0.12	0.23	0.04	0.06	0.07	0.03	0.14
**H2D**	H20	0.13	0.14	0.05	0.10	0.11	0.23	0.04	0.12	0.10	0.03	0.14
**C1**	C21	-0.16	-0.25	0.00	-0.11	-0.05	-0.23	0.35	-0.02	-0.13	-0.07	-0.01
**H1B**	H22	0.15	0.15	0.04	0.11	0.11	0.25	0.07	0.12	0.14	0.11	0.08
**H1A**	H23	0.11	0.13	0.03	0.09	0.10	0.19	0.01	0.05	0.07	0.05	0.08

Table S15 Ciprofloxacin: ωB97XD/6-31g.

**Atom**	**AtomQM**	**Mulliken**	**Loewdin**	**Hirshfeld**	**ADCH**	**CM5**	**NPA**	**AIM**	**Becke**	**MK**	**CHELPG**	**RESP**
**Cl1**	Cl1	-0.61	-0.62	-0.50	-0.50	-0.52	-0.85	-0.74	-0.80	-0.63	-0.62	-0.63
**C18**	C36	0.64	0.20	0.20	0.25	0.26	0.83	1.74	0.57	0.76	0.73	0.74
**O1**	O4	-0.50	-0.29	-0.30	-0.37	-0.35	-0.62	-1.24	-0.57	-0.52	-0.52	-0.52
**O3**	O5	-0.53	-0.23	-0.20	-0.29	-0.38	-0.67	-1.22	-0.55	-0.60	-0.60	-0.59
**H3**	H6	0.37	0.23	0.13	0.24	0.33	0.49	0.66	0.50	0.44	0.44	0.44
**C13**	C33	-0.20	-0.16	-0.07	-0.08	-0.06	-0.33	-0.04	-0.43	-0.47	-0.40	-0.43
**C12**	C34	0.13	0.02	0.06	-0.01	0.08	0.11	0.52	0.37	0.06	0.10	0.03
**H12**	H35	0.20	0.15	0.07	0.15	0.14	0.28	0.11	0.21	0.13	0.10	0.13
**N3**	N11	-0.58	0.00	0.00	0.02	-0.25	-0.38	-1.34	-0.27	0.01	-0.08	0.04
**C7**	C29	0.32	0.06	0.05	0.01	0.11	0.20	0.42	0.12	0.08	0.14	0.05
**C8**	C28	-0.02	-0.06	-0.03	-0.04	-0.03	-0.16	-0.01	0.05	-0.12	-0.12	-0.09
**C14**	C32	0.45	0.15	0.12	0.18	0.16	0.52	0.99	0.39	0.59	0.54	0.55
**O2**	O3	-0.59	-0.32	-0.26	-0.31	-0.33	-0.65	-1.21	-0.63	-0.56	-0.57	-0.55
**C15**	C37	-0.01	-0.06	0.01	-0.10	0.01	-0.10	0.32	-0.08	0.09	0.14	0.12
**H15**	H38	0.16	0.12	0.05	0.12	0.12	0.27	0.03	0.05	0.07	0.05	0.05
**C17**	C39	-0.29	-0.21	-0.06	-0.20	-0.16	-0.47	0.02	-0.18	-0.54	-0.40	-0.56
**H17B**	H40	0.16	0.13	0.06	0.15	0.11	0.27	0.03	0.09	0.21	0.16	0.22
**H17A**	H41	0.17	0.13	0.06	0.14	0.11	0.27	0.03	0.12	0.22	0.18	0.22
**C16**	C42	-0.26	-0.22	-0.07	-0.22	-0.17	-0.50	0.01	-0.19	-0.05	-0.04	0.00
**H16B**	H43	0.18	0.14	0.05	0.12	0.11	0.27	0.05	0.15	0.12	0.08	0.09
**H16A**	H44	0.17	0.13	0.05	0.13	0.11	0.26	0.03	0.09	0.10	0.08	0.09
**C6**	C30	-0.15	-0.17	-0.07	0.03	-0.11	-0.32	-0.01	0.55	-0.28	-0.29	-0.25
**H6**	H31	0.12	0.13	0.04	0.06	0.11	0.25	0.03	-0.13	0.13	0.12	0.12
**C5**	C24	0.21	0.04	0.04	-0.03	0.10	0.15	0.42	-0.10	0.21	0.29	0.19
**C10**	C25	0.34	0.12	0.09	0.18	0.11	0.37	0.52	-0.05	0.20	0.11	0.21
**F1**	F2	-0.29	-0.13	-0.09	-0.11	-0.12	-0.34	-0.66	-0.14	-0.16	-0.15	-0.16
**C9**	C26	-0.20	-0.10	-0.04	-0.22	-0.08	-0.22	0.04	-0.12	-0.23	-0.16	-0.24
**H9**	H27	0.19	0.15	0.07	0.19	0.13	0.30	0.09	0.20	0.20	0.18	0.20
**N1**	N7	-0.49	-0.14	-0.08	-0.19	-0.34	-0.47	-1.11	-0.33	-0.28	-0.36	-0.28
**C4**	C12	-0.11	-0.13	0.00	-0.10	-0.05	-0.30	0.42	0.02	-0.22	-0.07	-0.21
**H4B**	H13	0.14	0.12	0.04	0.10	0.11	0.25	0.00	0.06	0.13	0.07	0.14
**H4A**	H14	0.16	0.13	0.05	0.13	0.12	0.27	0.02	0.10	0.16	0.09	0.14
**C3**	C15	-0.12	-0.14	0.00	-0.12	-0.05	-0.29	0.33	-0.04	0.01	0.11	-0.02
**H3A**	H16	0.18	0.13	0.04	0.08	0.11	0.28	0.05	0.15	0.04	0.01	0.08
**H3B**	H17	0.17	0.13	0.06	0.14	0.12	0.28	0.03	0.08	0.09	0.03	0.08
**N2**	N8	-0.53	-0.10	0.00	0.02	-0.47	-0.65	-1.11	-0.34	0.02	0.15	0.01
**H2A**	H9	0.28	0.18	0.08	-0.03	0.23	0.47	0.50	0.49	0.09	0.04	0.10
**H2B**	H10	0.32	0.21	0.15	0.25	0.34	0.45	0.42	0.24	0.24	0.16	0.24
**C2**	C18	-0.12	-0.13	0.01	-0.12	-0.05	-0.30	0.34	-0.03	-0.01	0.10	-0.12
**H2C**	H19	0.17	0.13	0.06	0.14	0.12	0.28	0.03	0.07	0.08	0.03	0.13
**H2D**	H20	0.19	0.14	0.05	0.11	0.12	0.28	0.05	0.15	0.11	0.04	0.13
**C1**	C21	-0.12	-0.13	-0.01	-0.11	-0.05	-0.30	0.41	-0.01	-0.20	-0.09	-0.10
**H1B**	H22	0.18	0.14	0.05	0.12	0.12	0.29	0.05	0.12	0.16	0.12	0.11
**H1A**	H23	0.14	0.11	0.04	0.10	0.11	0.24	-0.01	0.06	0.12	0.07	0.11

Table S16 Ciprofloxacin: ωB97XD/6-311g.

Atom	AtomQM	Mulliken	Loewdin	Hirshfeld	ADCH	CM5	NPA	AIM	Becke	MK	CHELPG	RESP
**Cl1**	Cl1	-0.66	-0.54	-0.51	-0.45	-0.53	-0.87	-0.75	-0.75	-0.67	-0.65	-0.67
**C18**	C36	0.48	-0.08	0.20	0.27	0.26	0.81	1.64	0.58	0.83	0.78	0.81
**O1**	O4	-0.36	-0.10	-0.30	-0.37	-0.34	-0.61	-1.17	-0.57	-0.55	-0.54	-0.54
**O3**	O5	-0.32	0.08	-0.19	-0.29	-0.38	-0.64	-1.16	-0.56	-0.62	-0.61	-0.61
**H3**	H6	0.27	0.01	0.13	0.25	0.32	0.46	0.64	0.44	0.45	0.45	0.45
**C13**	C33	-0.52	-0.17	-0.07	-0.11	-0.06	-0.32	-0.05	-0.42	-0.56	-0.43	-0.52
**C12**	C34	0.26	0.09	0.06	0.00	0.08	0.14	0.46	0.40	0.08	0.12	0.05
**H12**	H35	0.18	0.03	0.07	0.14	0.14	0.24	0.13	0.21	0.13	0.10	0.14
**N3**	N11	-0.49	0.16	0.01	0.04	-0.25	-0.39	-1.24	-0.32	0.02	-0.09	0.05
**C7**	C29	0.32	0.01	0.05	0.03	0.11	0.21	0.39	0.23	0.11	0.14	0.07
**C8**	C28	-0.24	-0.07	-0.03	-0.04	-0.03	-0.16	-0.01	-0.01	-0.17	-0.13	-0.13
**C14**	C32	0.46	-0.02	0.12	0.19	0.16	0.52	0.94	0.43	0.66	0.57	0.63
**O2**	O3	-0.42	-0.12	-0.26	-0.32	-0.33	-0.66	-1.16	-0.57	-0.58	-0.58	-0.58
**C15**	C37	-0.12	0.00	0.01	-0.09	0.01	-0.04	0.29	-0.08	0.06	0.14	0.09
**H15**	H38	0.17	0.01	0.05	0.12	0.11	0.22	0.04	0.05	0.08	0.04	0.06
**C17**	C39	-0.25	0.01	-0.06	-0.19	-0.16	-0.39	-0.03	-0.17	-0.55	-0.38	-0.58
**H17B**	H40	0.16	0.02	0.06	0.14	0.11	0.22	0.05	0.08	0.22	0.15	0.22
**H17A**	H41	0.17	0.02	0.06	0.13	0.11	0.23	0.05	0.12	0.22	0.17	0.22
**C16**	C42	-0.24	0.01	-0.06	-0.21	-0.17	-0.42	-0.03	-0.19	-0.03	-0.03	0.02
**H16B**	H43	0.18	0.02	0.05	0.11	0.11	0.23	0.07	0.14	0.12	0.08	0.09
**H16A**	H44	0.17	0.02	0.05	0.12	0.10	0.22	0.06	0.08	0.11	0.08	0.09
**C6**	C30	-0.09	-0.05	-0.07	-0.09	-0.11	-0.30	-0.02	0.41	-0.31	-0.29	-0.28
**H6**	H31	0.15	0.01	0.04	0.09	0.11	0.22	0.04	-0.09	0.14	0.12	0.13
**C5**	C24	0.07	-0.03	0.04	0.01	0.10	0.17	0.40	0.03	0.19	0.27	0.17
**C10**	C25	0.28	-0.01	0.08	0.27	0.10	0.38	0.48	-0.24	0.24	0.14	0.24
**F1**	F2	-0.23	0.02	-0.09	-0.16	-0.12	-0.36	-0.63	-0.08	-0.19	-0.18	-0.19
**C9**	C26	-0.07	0.02	-0.04	-0.24	-0.08	-0.18	0.03	-0.09	-0.23	-0.16	-0.24
**H9**	H27	0.15	0.03	0.07	0.18	0.13	0.26	0.11	0.21	0.21	0.18	0.21
**N1**	N7	-0.39	0.04	-0.08	-0.19	-0.34	-0.48	-1.07	-0.33	-0.25	-0.35	-0.25
**C4**	C12	-0.17	0.01	0.00	-0.10	-0.05	-0.19	0.36	0.02	-0.22	-0.04	-0.20
**H4B**	H13	0.16	0.02	0.04	0.10	0.11	0.20	0.02	0.06	0.13	0.07	0.13
**H4A**	H14	0.17	0.02	0.05	0.12	0.12	0.22	0.05	0.10	0.16	0.08	0.13
**C3**	C15	-0.13	0.04	0.01	-0.12	-0.05	-0.19	0.28	-0.04	0.01	0.07	-0.03
**H3A**	H16	0.17	0.01	0.04	0.08	0.11	0.23	0.06	0.15	0.05	0.04	0.09
**H3B**	H17	0.17	0.03	0.06	0.13	0.12	0.23	0.05	0.07	0.08	0.04	0.09
**N2**	N8	-0.35	0.27	0.01	0.15	-0.47	-0.60	-1.04	-0.22	0.07	0.16	0.05
**H2A**	H9	0.26	-0.03	0.07	-0.21	0.23	0.47	0.49	0.32	0.09	0.06	0.10
**H2B**	H10	0.26	0.06	0.15	0.25	0.34	0.41	0.39	0.24	0.22	0.16	0.23
**C2**	C18	-0.14	0.04	0.01	-0.12	-0.04	-0.19	0.28	-0.03	-0.02	0.07	-0.14
**H2C**	H19	0.17	0.03	0.06	0.14	0.12	0.23	0.05	0.07	0.08	0.04	0.13
**H2D**	H20	0.19	0.03	0.05	0.10	0.12	0.23	0.07	0.15	0.11	0.04	0.13
**C1**	C21	-0.15	0.02	-0.01	-0.11	-0.05	-0.20	0.36	0.00	-0.21	-0.08	-0.11
**H1B**	H22	0.18	0.02	0.05	0.12	0.12	0.24	0.07	0.13	0.17	0.12	0.12
**H1A**	H23	0.14	0.01	0.04	0.10	0.11	0.19	0.01	0.06	0.12	0.07	0.12

Table S17 Ciprofloxacin: ωB97XD/def2tzvp.

Atom	AtomQM	Mulliken	Loewdin	Hirshfeld	ADCH	CM5	NPA	AIM	Becke	MK	CHELPG	RESP
**Cl1**	Cl1	-0.65	-0.54	-0.52	-0.52	-0.54	-0.86	-0.77	-0.79	-0.63	-0.61	-0.63
**C18**	C36	0.37	-0.50	0.19	0.46	0.25	0.76	1.70	0.54	0.79	0.78	0.77
**O1**	O4	-0.37	0.09	-0.30	-0.46	-0.35	-0.59	-1.22	-0.58	-0.55	-0.55	-0.55
**O3**	O5	-0.38	0.13	-0.19	-0.39	-0.38	-0.62	-1.19	-0.52	-0.60	-0.60	-0.59
**H3**	H6	0.35	0.18	0.12	0.30	0.32	0.47	0.65	0.38	0.44	0.44	0.44
**C13**	C33	-0.19	-0.14	-0.07	-0.14	-0.06	-0.34	-0.06	-0.13	-0.47	-0.41	-0.44
**C12**	C34	-0.02	-0.07	0.06	-0.03	0.08	0.14	0.48	0.22	0.05	0.10	0.03
**H12**	H35	0.21	0.17	0.07	0.15	0.14	0.25	0.13	0.28	0.13	0.10	0.13
**N3**	N11	0.06	0.29	0.01	0.04	-0.25	-0.32	-1.24	-0.30	0.06	-0.06	0.08
**C7**	C29	-0.05	-0.11	0.05	0.03	0.11	0.19	0.38	0.24	0.05	0.13	0.02
**C8**	C28	0.08	-0.08	-0.03	-0.05	-0.02	-0.17	-0.01	0.10	-0.09	-0.11	-0.06
**C14**	C32	0.23	-0.28	0.12	0.31	0.16	0.50	0.97	0.09	0.58	0.54	0.55
**O2**	O3	-0.48	0.06	-0.26	-0.42	-0.33	-0.63	-1.20	-0.48	-0.58	-0.58	-0.57
**C15**	C37	-0.14	-0.23	0.01	-0.10	0.01	-0.08	0.28	-0.07	0.09	0.13	0.11
**H15**	H38	0.13	0.15	0.05	0.12	0.11	0.22	0.05	0.05	0.06	0.04	0.05
**C17**	C39	-0.20	-0.20	-0.06	-0.19	-0.16	-0.41	-0.04	-0.17	-0.52	-0.36	-0.55
**H17B**	H40	0.14	0.14	0.06	0.14	0.11	0.23	0.06	0.08	0.21	0.15	0.21
**H17A**	H41	0.16	0.14	0.06	0.13	0.11	0.24	0.06	0.12	0.21	0.16	0.21
**C16**	C42	-0.28	-0.21	-0.06	-0.22	-0.17	-0.43	-0.05	-0.19	-0.03	-0.03	0.03
**H16B**	H43	0.16	0.15	0.05	0.12	0.11	0.24	0.07	0.14	0.10	0.07	0.08
**H16A**	H44	0.16	0.14	0.05	0.13	0.11	0.22	0.06	0.08	0.10	0.08	0.08
**C6**	C30	-0.20	-0.09	-0.07	-0.07	-0.11	-0.30	-0.03	0.39	-0.29	-0.28	-0.27
**H6**	H31	0.14	0.16	0.04	0.08	0.11	0.22	0.05	-0.09	0.14	0.13	0.13
**C5**	C24	0.01	-0.17	0.04	0.02	0.10	0.15	0.41	0.04	0.19	0.24	0.18
**C10**	C25	0.36	-0.24	0.08	0.19	0.10	0.32	0.53	-0.34	0.23	0.13	0.23
**F1**	F2	-0.21	0.20	-0.09	-0.12	-0.12	-0.32	-0.67	-0.01	-0.19	-0.18	-0.19
**C9**	C26	-0.31	-0.05	-0.03	-0.28	-0.08	-0.18	0.02	-0.08	-0.24	-0.14	-0.24
**H9**	H27	0.17	0.17	0.06	0.22	0.13	0.26	0.12	0.22	0.20	0.17	0.20
**N1**	N7	-0.07	0.17	-0.08	-0.16	-0.34	-0.40	-1.08	-0.32	-0.26	-0.31	-0.26
**C4**	C12	-0.17	-0.27	-0.01	-0.11	-0.05	-0.23	0.36	0.01	-0.13	0.00	-0.13
**H4B**	H13	0.15	0.14	0.04	0.10	0.11	0.21	0.02	0.06	0.11	0.05	0.12
**H4A**	H14	0.16	0.15	0.05	0.13	0.12	0.23	0.05	0.10	0.13	0.07	0.12
**C3**	C15	-0.27	-0.27	0.01	-0.13	-0.05	-0.24	0.27	-0.05	-0.06	0.08	-0.10
**H3A**	H16	0.17	0.14	0.04	0.08	0.11	0.23	0.07	0.15	0.05	0.01	0.10
**H3B**	H17	0.17	0.15	0.06	0.14	0.12	0.24	0.06	0.08	0.10	0.03	0.10
**N2**	N8	-0.16	0.15	0.01	0.01	-0.48	-0.54	-1.04	-0.32	0.06	0.20	0.04
**H2A**	H9	0.28	0.11	0.07	-0.01	0.24	0.48	0.51	0.46	0.06	0.00	0.07
**H2B**	H10	0.25	0.20	0.15	0.26	0.34	0.41	0.40	0.25	0.24	0.16	0.25
**C2**	C18	-0.19	-0.26	0.01	-0.12	-0.04	-0.24	0.27	-0.04	-0.05	0.09	-0.14
**H2C**	H19	0.16	0.14	0.06	0.14	0.13	0.23	0.06	0.07	0.09	0.04	0.13
**H2D**	H20	0.17	0.15	0.05	0.11	0.12	0.24	0.07	0.15	0.12	0.04	0.13
**C1**	C21	-0.21	-0.26	-0.01	-0.12	-0.05	-0.24	0.35	-0.01	-0.19	-0.11	-0.11
**H1B**	H22	0.17	0.15	0.05	0.12	0.12	0.25	0.07	0.13	0.16	0.13	0.12
**H1A**	H23	0.14	0.14	0.04	0.10	0.11	0.20	0.02	0.05	0.12	0.07	0.12

S1.7.2 Tyrosine

Table S18 Tyrosine: B3LYP/6-31g.

Atom	AtomQM	Mulliken	Loewdin	Hirshfeld	ADCH	CM5	NPA	AIM	Becke	MK	CHELPG	RESP	PEOE
**C1**	C4	0.65	-0.15	-0.02	0.41	-0.03	-0.09	-0.01	0.99	0.10	0.08	0.09	-0.05
**C2**	C5	-0.41	-0.16	-0.04	-0.74	-0.09	-0.22	0.00	-1.72	-0.20	-0.17	-0.19	-0.06
**H2**	H9	0.13	0.20	0.05	0.59	0.10	0.25	0.01	1.15	0.16	0.13	0.16	0.06
**C3**	C6	0.30	-0.16	-0.05	-0.69	-0.10	-0.27	0.01	-0.12	-0.27	-0.19	-0.27	-0.03
**H3**	H10	0.14	0.21	0.05	0.61	0.11	0.26	0.03	0.28	0.19	0.15	0.19	0.07
**C4**	C1	-0.21	0.08	0.08	0.26	0.09	0.31	0.52	0.67	0.48	0.44	0.48	0.07
**O2**	O11	-0.51	-0.38	-0.19	-0.59	-0.39	-0.70	-1.14	-0.74	-0.62	-0.63	-0.61	-0.36
**H2A**	H12	0.36	0.30	0.17	0.54	0.35	0.51	0.59	0.52	0.43	0.43	0.43	0.22
**C5**	C2	0.23	-0.20	-0.07	-0.67	-0.12	-0.31	-0.01	-0.52	-0.40	-0.32	-0.40	-0.03
**H5**	H7	0.12	0.19	0.04	0.51	0.10	0.24	0.00	0.37	0.17	0.13	0.17	0.07
**C6**	C3	-0.60	-0.19	-0.05	-0.58	-0.10	-0.24	-0.01	-1.70	-0.11	-0.09	-0.10	-0.06
**H6**	H8	0.13	0.20	0.04	0.48	0.10	0.24	0.00	1.15	0.13	0.11	0.13	0.06
**C7**	C13	-0.56	-0.27	-0.05	-0.25	-0.14	-0.49	0.08	-0.53	-0.18	-0.10	-0.16	0.00
**H7A**	H14	0.16	0.20	0.04	0.10	0.10	0.26	-0.01	0.11	0.09	0.05	0.10	0.03
**H7B**	H15	0.21	0.22	0.05	0.13	0.11	0.30	0.02	0.14	0.11	0.07	0.10	0.03
**C8**	C16	-0.04	-0.14	0.03	0.12	0.02	-0.20	0.37	-0.12	0.04	0.12	0.01	0.10
**H8**	H17	0.17	0.20	0.04	0.10	0.11	0.27	0.01	0.09	0.04	0.01	0.06	0.06
**N1**	N21	-0.58	-0.24	0.02	-0.14	-0.58	-0.80	-1.10	-0.29	-0.35	-0.30	-0.33	-0.32
**H1A**	H22	0.37	0.28	0.17	0.26	0.35	0.49	0.62	0.26	0.29	0.27	0.29	0.22
**H1B**	H23	0.40	0.31	0.13	0.21	0.33	0.46	0.39	0.31	0.31	0.30	0.30	0.12
**H1C**	H24	0.35	0.28	0.14	0.20	0.32	0.46	0.38	0.28	0.21	0.17	0.21	0.12
**C9**	C18	0.29	0.17	0.13	-0.37	0.19	0.75	1.62	1.29	0.78	0.78	0.78	0.28
**O1**	O19	-0.58	-0.50	-0.36	-0.24	-0.42	-0.77	-1.17	-0.95	-0.72	-0.73	-0.72	-0.33
**O3**	O20	-0.53	-0.45	-0.38	-0.24	-0.42	-0.71	-1.21	-0.92	-0.70	-0.70	-0.70	-0.26

Table S19 Tyrosine: B3LYP/6-311g.

Atom	AtomQM	Mulliken	Loewdin	Hirshfeld	ADCH	CM5	NPA	AIM	Becke	MK	CHELPG	RESP	PEOE
**C1**	C4	1.35	-0.15	-0.01	0.44	-0.03	-0.09	-0.04	1.02	0.11	0.08	0.09	-0.04
**C2**	C5	-0.71	-0.05	-0.04	-0.78	-0.09	-0.18	-0.02	-1.80	-0.20	-0.16	-0.20	-0.06
**H2**	H9	0.13	0.09	0.05	0.60	0.10	0.21	0.03	1.19	0.17	0.13	0.17	0.06
**C3**	C6	0.15	-0.05	-0.05	-0.67	-0.10	-0.24	0.00	-0.10	-0.29	-0.21	-0.29	-0.03
**H3**	H10	0.14	0.09	0.05	0.60	0.11	0.23	0.05	0.29	0.19	0.15	0.19	0.07
**C4**	C1	-0.38	-0.03	0.07	0.29	0.09	0.32	0.49	0.72	0.50	0.44	0.49	0.07
**O2**	O11	-0.25	-0.09	-0.18	-0.58	-0.38	-0.67	-1.09	-0.73	-0.61	-0.62	-0.61	-0.36
**H2A**	H12	0.25	0.12	0.17	0.53	0.34	0.47	0.57	0.51	0.42	0.43	0.42	0.22
**C5**	C2	0.10	-0.08	-0.07	-0.68	-0.12	-0.28	-0.03	-0.55	-0.42	-0.33	-0.42	-0.03
**H5**	H7	0.12	0.08	0.04	0.51	0.10	0.21	0.02	0.37	0.18	0.13	0.18	0.07
**C6**	C3	-0.55	-0.08	-0.05	-0.67	-0.10	-0.21	-0.04	-1.85	-0.11	-0.08	-0.10	-0.06
**H6**	H8	0.13	0.08	0.04	0.53	0.10	0.21	0.02	1.26	0.14	0.11	0.13	0.06
**C7**	C13	-0.83	-0.08	-0.05	-0.25	-0.14	-0.41	0.04	-0.53	-0.21	-0.12	-0.19	0.03
**H7A**	H14	0.17	0.10	0.04	0.10	0.10	0.22	0.01	0.11	0.10	0.06	0.11	0.04
**H7B**	H15	0.22	0.11	0.05	0.13	0.11	0.26	0.06	0.14	0.12	0.07	0.11	0.04
**C8**	C16	-0.08	-0.06	0.03	0.10	0.01	-0.13	0.22	-0.14	0.04	0.14	0.01	0.08
**H8**	H17	0.18	0.09	0.04	0.10	0.11	0.23	0.03	0.09	0.05	0.00	0.06	0.10
**N1**	N21	-0.27	0.20	0.02	-0.14	-0.58	-0.68	-0.98	-0.29	-0.37	-0.33	-0.35	0.24
**H1A**	H22	0.30	0.15	0.17	0.26	0.35	0.42	0.41	0.26	0.30	0.27	0.29	0.20
**H1B**	H23	0.33	0.15	0.13	0.22	0.33	0.46	0.50	0.31	0.32	0.31	0.31	0.20
**H1C**	H24	0.30	0.14	0.14	0.20	0.32	0.41	0.41	0.28	0.22	0.18	0.21	0.20
**C9**	C18	0.00	-0.12	0.13	-0.32	0.19	0.72	1.70	1.37	0.79	0.78	0.79	0.40
**O1**	O19	-0.42	-0.33	-0.36	-0.25	-0.42	-0.77	-1.21	-0.98	-0.72	-0.73	-0.72	-0.24
**O3**	O20	-0.37	-0.29	-0.37	-0.26	-0.42	-0.71	-1.17	-0.95	-0.70	-0.70	-0.70	-0.24

Table S20 Tyrosine: B3LYP/def2tzvp.

Atom	AtomQM	Mulliken	Loewdin	Hirshfeld	ADCH	CM5	NPA	AIM	Becke	MK	CHELPG	RESP	PEOE
**C1**	C4	0.12	-0.17	-0.01	0.42	-0.03	-0.08	-0.04	0.98	0.14	0.12	0.12	-0.04
**C2**	C5	-0.17	-0.10	-0.04	-0.74	-0.09	-0.18	-0.03	-1.71	-0.22	-0.19	-0.21	-0.06
**H2**	H9	0.13	0.15	0.05	0.60	0.10	0.22	0.04	1.18	0.17	0.13	0.16	0.06
**C3**	C6	-0.18	-0.11	-0.05	-0.66	-0.10	-0.24	-0.01	-0.28	-0.25	-0.18	-0.25	-0.03
**H3**	H10	0.12	0.15	0.05	0.59	0.11	0.23	0.06	0.33	0.18	0.14	0.18	0.07
**C4**	C1	0.27	-0.27	0.08	0.21	0.09	0.29	0.51	0.79	0.45	0.40	0.44	0.07
**O2**	O11	-0.39	0.09	-0.18	-0.55	-0.38	-0.64	-1.11	-0.63	-0.57	-0.58	-0.57	-0.36
**H2A**	H12	0.31	0.19	0.17	0.54	0.35	0.47	0.58	0.42	0.41	0.42	0.41	0.22
**C5**	C2	-0.18	-0.14	-0.07	-0.64	-0.12	-0.28	-0.04	-0.63	-0.40	-0.32	-0.40	-0.03
**H5**	H7	0.11	0.14	0.04	0.50	0.10	0.21	0.03	0.41	0.17	0.13	0.17	0.07
**C6**	C3	-0.24	-0.14	-0.05	-0.69	-0.10	-0.21	-0.06	-1.75	-0.11	-0.08	-0.11	-0.06
**H6**	H8	0.13	0.15	0.04	0.54	0.10	0.21	0.03	1.22	0.13	0.10	0.13	0.06
**C7**	C13	-0.28	-0.20	-0.05	-0.26	-0.14	-0.42	0.04	-0.53	-0.24	-0.17	-0.21	0.03
**H7A**	H14	0.13	0.15	0.04	0.10	0.10	0.21	0.02	0.11	0.10	0.07	0.11	0.04
**H7B**	H15	0.15	0.17	0.05	0.13	0.11	0.26	0.07	0.14	0.12	0.08	0.11	0.04
**C8**	C16	-0.11	-0.23	0.03	0.09	0.01	-0.16	0.20	-0.17	0.04	0.12	0.01	0.08
**H8**	H17	0.12	0.15	0.04	0.10	0.11	0.22	0.04	0.09	0.05	0.01	0.06	0.10
**N1**	N21	-0.25	0.16	0.01	-0.15	-0.58	-0.66	-1.00	-0.30	-0.35	-0.31	-0.33	0.24
**H1A**	H22	0.28	0.20	0.17	0.26	0.35	0.42	0.42	0.25	0.29	0.27	0.29	0.20
**H1B**	H23	0.29	0.19	0.13	0.21	0.33	0.46	0.51	0.30	0.31	0.30	0.30	0.20
**H1C**	H24	0.26	0.19	0.14	0.20	0.32	0.41	0.42	0.28	0.21	0.17	0.21	0.20
**C9**	C18	0.29	-0.66	0.13	-0.32	0.20	0.70	1.78	1.47	0.72	0.72	0.72	0.40
**O1**	O19	-0.47	-0.04	-0.36	-0.23	-0.41	-0.75	-1.25	-0.99	-0.68	-0.69	-0.67	-0.24
**O3**	O20	-0.43	-0.01	-0.37	-0.25	-0.42	-0.68	-1.21	-0.99	-0.66	-0.67	-0.66	-0.24

Table S21 Tyrosine: ωB97XD/6-31g.

Atom	AtomQM	Mulliken	Loewdin	Hirshfeld	ADCH	CM5	NPA	AIM	Becke	MK	CHELPG	RESP	PEOE
**C1**	C4	0.48	-0.15	-0.02	0.51	-0.03	-0.10	-0.03	1.03	0.12	0.10	0.11	-0.04
**C2**	C5	-0.38	-0.16	-0.04	-0.49	-0.09	-0.22	-0.01	-1.12	-0.21	-0.18	-0.20	-0.06
**H2**	H9	0.16	0.21	0.05	0.46	0.11	0.26	0.02	0.81	0.17	0.14	0.17	0.06
**C3**	C6	0.33	-0.17	-0.05	-0.71	-0.10	-0.28	0.01	-0.23	-0.29	-0.21	-0.28	-0.03
**H3**	H10	0.18	0.21	0.06	0.68	0.11	0.27	0.04	0.31	0.20	0.16	0.20	0.07
**C4**	C1	-0.30	0.08	0.08	-0.03	0.09	0.32	0.55	0.33	0.48	0.45	0.48	0.07
**O2**	O11	-0.52	-0.38	-0.19	-0.65	-0.39	-0.71	-1.17	-0.78	-0.62	-0.63	-0.62	-0.36
**H2A**	H12	0.37	0.30	0.18	0.57	0.35	0.51	0.61	0.55	0.44	0.44	0.43	0.22
**C5**	C2	0.22	-0.21	-0.07	-0.41	-0.12	-0.32	-0.02	-0.40	-0.42	-0.35	-0.41	-0.03
**H5**	H7	0.15	0.20	0.05	0.59	0.10	0.25	0.01	0.43	0.19	0.15	0.18	0.07
**C6**	C3	-0.59	-0.20	-0.05	-0.82	-0.10	-0.25	-0.03	-1.23	-0.11	-0.08	-0.11	-0.06
**H6**	H8	0.16	0.20	0.05	0.45	0.10	0.25	0.01	0.72	0.14	0.11	0.14	0.06
**C7**	C13	-0.53	-0.28	-0.05	-0.29	-0.15	-0.51	0.07	-0.62	-0.27	-0.19	-0.25	0.03
**H7A**	H14	0.18	0.21	0.04	0.11	0.10	0.27	0.00	0.12	0.12	0.08	0.13	0.04
**H7B**	H15	0.23	0.22	0.06	0.14	0.12	0.31	0.05	0.14	0.14	0.10	0.13	0.04
**C8**	C16	-0.05	-0.15	0.03	0.07	0.01	-0.20	0.26	-0.10	0.03	0.12	0.00	0.08
**H8**	H17	0.19	0.21	0.05	0.11	0.12	0.28	0.02	0.09	0.06	0.02	0.07	0.10
**N1**	N21	-0.58	-0.24	0.01	-0.14	-0.58	-0.81	-1.16	-0.30	-0.39	-0.33	-0.36	0.24
**H1A**	H22	0.38	0.28	0.18	0.27	0.36	0.48	0.46	0.27	0.31	0.28	0.31	0.20
**H1B**	H23	0.41	0.31	0.14	0.22	0.34	0.50	0.55	0.32	0.33	0.32	0.32	0.20
**H1C**	H24	0.36	0.29	0.14	0.20	0.33	0.46	0.46	0.28	0.23	0.19	0.22	0.20
**C9**	C18	0.29	0.18	0.13	-0.24	0.20	0.76	1.87	1.18	0.78	0.77	0.78	0.40
**O1**	O19	-0.60	-0.51	-0.37	-0.29	-0.43	-0.79	-1.30	-0.91	-0.73	-0.73	-0.72	-0.24
**O3**	O20	-0.54	-0.46	-0.39	-0.30	-0.43	-0.72	-1.26	-0.89	-0.71	-0.70	-0.70	-0.24

Table S22 Tyrosine: ωB97XD/6-311g.

Atom	AtomQM	Mulliken	Loewdin	Hirshfeld	ADCH	CM5	NPA	AIM	Becke	MK	CHELPG	RESP	PEOE
**C1**	C4	1.28	-0.15	-0.02	0.57	-0.03	-0.09	-0.04	1.19	0.12	0.10	0.11	-0.04
**C2**	C5	-0.71	-0.05	-0.04	-0.56	-0.09	-0.18	-0.03	-1.25	-0.21	-0.19	-0.20	-0.06
**H2**	H9	0.16	0.09	0.05	0.50	0.11	0.22	0.04	0.93	0.18	0.14	0.18	0.06
**C3**	C6	0.15	-0.05	-0.05	-0.69	-0.10	-0.24	-0.01	-0.48	-0.30	-0.21	-0.30	-0.03
**H3**	H10	0.17	0.09	0.05	0.68	0.11	0.23	0.06	0.48	0.20	0.16	0.20	0.07
**C4**	C1	-0.39	-0.03	0.07	0.02	0.09	0.32	0.52	0.42	0.50	0.44	0.49	0.07
**O2**	O11	-0.25	-0.08	-0.19	-0.64	-0.39	-0.67	-1.12	-0.78	-0.61	-0.63	-0.61	-0.36
**H2A**	H12	0.26	0.12	0.18	0.56	0.35	0.47	0.58	0.54	0.43	0.43	0.43	0.22
**C5**	C2	0.09	-0.09	-0.07	-0.48	-0.12	-0.29	-0.03	-0.38	-0.44	-0.35	-0.43	-0.03
**H5**	H7	0.14	0.08	0.04	0.63	0.10	0.21	0.02	0.46	0.19	0.15	0.19	0.07
**C6**	C3	-0.63	-0.08	-0.05	-0.96	-0.10	-0.21	-0.05	-1.61	-0.11	-0.08	-0.11	-0.06
**H6**	H8	0.16	0.09	0.04	0.53	0.10	0.21	0.03	0.95	0.14	0.11	0.14	0.06
**C7**	C13	-0.88	-0.09	-0.05	-0.30	-0.15	-0.42	0.03	-0.66	-0.28	-0.18	-0.26	0.03
**H7A**	H14	0.20	0.10	0.04	0.11	0.10	0.22	0.02	0.12	0.12	0.08	0.13	0.04
**H7B**	H15	0.25	0.11	0.06	0.13	0.12	0.26	0.07	0.14	0.14	0.09	0.13	0.04
**C8**	C16	-0.10	-0.06	0.02	0.05	0.01	-0.13	0.23	-0.12	0.02	0.13	-0.01	0.08
**H8**	H17	0.21	0.10	0.04	0.11	0.11	0.23	0.04	0.09	0.06	0.01	0.07	0.10
**N1**	N21	-0.31	0.21	0.01	-0.14	-0.59	-0.69	-1.03	-0.30	-0.39	-0.34	-0.37	0.24
**H1A**	H22	0.33	0.15	0.18	0.27	0.36	0.43	0.42	0.27	0.31	0.28	0.31	0.20
**H1B**	H23	0.35	0.15	0.14	0.22	0.34	0.46	0.51	0.32	0.33	0.32	0.32	0.20
**H1C**	H24	0.32	0.14	0.14	0.20	0.33	0.42	0.42	0.28	0.23	0.18	0.23	0.20
**C9**	C18	-0.01	-0.12	0.13	-0.19	0.20	0.74	1.76	1.23	0.80	0.77	0.79	0.40
**O1**	O19	-0.42	-0.33	-0.37	-0.31	-0.43	-0.78	-1.24	-0.93	-0.73	-0.73	-0.73	-0.24
**O3**	O20	-0.37	-0.30	-0.38	-0.32	-0.43	-0.72	-1.20	-0.90	-0.71	-0.70	-0.70	-0.24

Table S23 Tyrosine: ωB97XD/def2tzvp.

Atom	AtomQM	Mulliken	Loewdin	Hirshfeld	ADCH	CM5	NPA	AIM	Becke	MK	CHELPG	RESP	PEOE
**C1**	C4	0.12	-0.18	-0.02	0.52	-0.03	-0.08	-0.04	1.03	0.15	0.11	0.13	-0.04
**C2**	C5	-0.19	-0.11	-0.04	-0.47	-0.09	-0.18	-0.04	-1.02	-0.22	-0.19	-0.21	-0.06
**H2**	H9	0.14	0.16	0.05	0.46	0.11	0.22	0.05	0.79	0.17	0.14	0.17	0.06
**C3**	C6	-0.20	-0.11	-0.05	-0.66	-0.10	-0.25	-0.02	-0.50	-0.26	-0.19	-0.26	-0.03
**H3**	H10	0.14	0.15	0.05	0.64	0.11	0.23	0.07	0.45	0.19	0.15	0.19	0.07
**C4**	C1	0.29	-0.28	0.08	-0.09	0.09	0.30	0.54	0.30	0.44	0.41	0.44	0.07
**O2**	O11	-0.41	0.09	-0.19	-0.58	-0.39	-0.64	-1.15	-0.73	-0.57	-0.59	-0.57	-0.36
**H2A**	H12	0.33	0.19	0.18	0.55	0.35	0.48	0.60	0.52	0.42	0.42	0.42	0.22
**C5**	C2	-0.19	-0.14	-0.07	-0.36	-0.12	-0.29	-0.04	-0.28	-0.41	-0.33	-0.41	-0.03
**H5**	H7	0.12	0.15	0.04	0.57	0.10	0.21	0.03	0.39	0.18	0.14	0.18	0.07
**C6**	C3	-0.28	-0.14	-0.05	-0.90	-0.10	-0.21	-0.06	-1.30	-0.12	-0.08	-0.11	-0.06
**H6**	H8	0.15	0.15	0.04	0.48	0.10	0.21	0.03	0.74	0.14	0.11	0.14	0.06
**C7**	C13	-0.29	-0.20	-0.05	-0.30	-0.15	-0.43	0.03	-0.61	-0.30	-0.20	-0.27	0.03
**H7A**	H14	0.15	0.16	0.04	0.11	0.10	0.22	0.02	0.12	0.13	0.08	0.13	0.04
**H7B**	H15	0.17	0.17	0.06	0.14	0.12	0.27	0.07	0.15	0.14	0.10	0.13	0.04
**C8**	C16	-0.14	-0.24	0.02	0.04	0.01	-0.17	0.21	-0.15	0.01	0.08	-0.01	0.08
**H8**	H17	0.14	0.15	0.04	0.11	0.12	0.23	0.04	0.09	0.06	0.02	0.07	0.10
**N1**	N21	-0.27	0.16	0.01	-0.15	-0.59	-0.66	-1.06	-0.31	-0.37	-0.32	-0.35	0.24
**H1A**	H22	0.30	0.21	0.18	0.27	0.36	0.43	0.43	0.27	0.31	0.28	0.30	0.20
**H1B**	H23	0.31	0.20	0.14	0.22	0.34	0.46	0.52	0.31	0.32	0.31	0.31	0.20
**H1C**	H24	0.27	0.20	0.14	0.20	0.33	0.42	0.43	0.28	0.22	0.18	0.22	0.20
**C9**	C18	0.34	-0.66	0.13	-0.22	0.20	0.72	1.84	1.32	0.72	0.72	0.72	0.40
**O1**	O19	-0.51	-0.05	-0.36	-0.28	-0.42	-0.76	-1.28	-0.95	-0.68	-0.70	-0.68	-0.24
**O3**	O20	-0.47	-0.02	-0.38	-0.30	-0.43	-0.70	-1.24	-0.94	-0.67	-0.67	-0.67	-0.24

S1.7.3 Histidine

Table S24 Histidine: B3LYP/6-31g.

Atom	AtomQM	Mulliken	Loewdin	Hirshfeld	ADCH	CM5	NPA	AIM	Becke	MK	CHELPG	RESP	PEOE
**C1**	C8	0.41	-0.07	0.01	0.02	0.08	0.05	0.41	0.19	0.33	0.30	0.31	0.06
**C2**	C6	-0.08	-0.13	-0.03	-0.12	0.00	-0.10	0.38	-0.02	-0.37	-0.27	-0.37	0.02
**H2**	H7	0.16	0.21	0.06	0.16	0.13	0.26	0.06	0.07	0.22	0.18	0.22	0.08
**N1**	N4	-0.34	-0.22	-0.04	-0.10	-0.39	-0.56	-1.30	-0.13	-0.12	-0.20	-0.12	-0.31
**H1**	H5	0.32	0.28	0.16	0.27	0.35	0.45	0.46	0.20	0.30	0.31	0.30	0.15
**C3**	C2	0.31	-0.02	0.05	-0.01	0.15	0.17	1.10	0.19	0.01	0.12	0.00	0.08
**H3**	H3	0.15	0.21	0.06	0.15	0.14	0.24	0.07	0.08	0.15	0.11	0.15	0.10
**N2**	N1	-0.34	-0.32	-0.18	-0.31	-0.37	-0.53	-1.24	-0.56	-0.37	-0.41	-0.36	-0.24
**C4**	C9	-0.94	-0.25	-0.05	-0.15	-0.14	-0.50	0.09	-0.17	-0.28	-0.17	-0.25	0.04
**H4A**	H10	0.20	0.21	0.05	0.12	0.11	0.30	0.05	0.12	0.12	0.08	0.12	0.04
**H4B**	H11	0.18	0.21	0.04	0.11	0.10	0.28	0.01	0.12	0.12	0.08	0.12	0.04
**C5**	C12	0.06	-0.13	0.03	0.10	0.02	-0.18	0.26	-0.10	0.08	0.11	0.07	0.08
**H5**	H13	0.17	0.20	0.04	0.10	0.11	0.26	0.02	0.09	0.03	-0.01	0.03	0.10
**N3**	N14	-0.61	-0.26	0.01	-0.12	-0.58	-0.80	-1.14	-0.30	-0.10	-0.10	-0.07	0.24
**H3A**	H16	0.36	0.28	0.17	0.25	0.34	0.46	0.43	0.26	0.22	0.22	0.22	0.20
**H3B**	H17	0.40	0.31	0.13	0.22	0.33	0.49	0.53	0.30	0.23	0.23	0.23	0.20
**H3C**	H15	0.40	0.29	0.11	0.17	0.31	0.47	0.51	0.26	0.11	0.10	0.10	0.20
**C6**	C18	0.33	0.17	0.13	-0.27	0.19	0.71	1.80	1.30	0.71	0.75	0.71	0.40
**O1**	O19	-0.54	-0.45	-0.38	-0.27	-0.42	-0.70	-1.25	-0.95	-0.68	-0.70	-0.68	-0.24
**O2**	O20	-0.59	-0.52	-0.38	-0.30	-0.43	-0.77	-1.25	-0.96	-0.71	-0.73	-0.71	-0.24

Table S25 Histidine: B3LYP/6-311g.

Atom	AtomQM	Mulliken	Loewdin	Hirshfeld	ADCH	CM5	NPA	AIM	Becke	MK	CHELPG	RESP	PEOE
**C1**	C8	0.52	-0.12	0.01	0.02	0.08	0.07	0.38	0.18	0.33	0.29	0.31	0.06
**C2**	C6	-0.09	-0.06	-0.03	-0.12	-0.01	-0.08	0.32	-0.02	-0.39	-0.28	-0.38	0.02
**H2**	H7	0.14	0.09	0.06	0.16	0.12	0.22	0.09	0.07	0.23	0.18	0.23	0.08
**N1**	N4	-0.18	0.04	-0.04	-0.10	-0.39	-0.52	-1.17	-0.12	-0.10	-0.18	-0.10	-0.31
**H1**	H5	0.31	0.12	0.15	0.27	0.34	0.41	0.43	0.20	0.29	0.30	0.29	0.15
**C3**	C2	0.20	-0.01	0.05	-0.01	0.15	0.20	0.94	0.19	-0.01	0.10	-0.02	0.08
**H3**	H3	0.13	0.09	0.06	0.15	0.13	0.20	0.08	0.08	0.15	0.11	0.15	0.10
**N2**	N1	-0.23	-0.18	-0.17	-0.31	-0.37	-0.54	-1.13	-0.55	-0.35	-0.39	-0.35	-0.24
**C4**	C9	-1.17	-0.07	-0.05	-0.15	-0.14	-0.42	0.05	-0.17	-0.30	-0.17	-0.27	0.04
**H4A**	H10	0.21	0.11	0.05	0.12	0.11	0.25	0.06	0.12	0.13	0.09	0.12	0.04
**H4B**	H11	0.20	0.10	0.04	0.10	0.10	0.23	0.03	0.12	0.13	0.08	0.12	0.04
**C5**	C12	-0.11	-0.05	0.03	0.09	0.01	-0.11	0.23	-0.11	0.08	0.10	0.06	0.08
**H5**	H13	0.19	0.09	0.04	0.10	0.11	0.21	0.03	0.09	0.03	0.00	0.03	0.10
**N3**	N14	-0.30	0.18	0.01	-0.12	-0.58	-0.69	-1.03	-0.30	-0.11	-0.10	-0.08	0.24
**H3A**	H16	0.30	0.15	0.17	0.25	0.34	0.42	0.41	0.25	0.22	0.22	0.22	0.20
**H3B**	H17	0.31	0.15	0.13	0.22	0.33	0.45	0.49	0.30	0.24	0.23	0.23	0.20
**H3C**	H15	0.35	0.13	0.11	0.17	0.31	0.44	0.48	0.26	0.11	0.10	0.10	0.20
**C6**	C18	0.03	-0.12	0.12	-0.24	0.19	0.70	1.68	1.33	0.71	0.76	0.71	0.40
**O1**	O19	-0.39	-0.29	-0.37	-0.28	-0.42	-0.70	-1.18	-0.95	-0.68	-0.70	-0.68	-0.24
**O2**	O20	-0.43	-0.35	-0.37	-0.31	-0.43	-0.78	-1.19	-0.97	-0.71	-0.73	-0.71	-0.24

Table S26 Histidine: B3LYP/def2tzvp.

**Atom**	**AtomQM**	**Mulliken**	**Loewdin**	**Hirshfeld**	**ADCH**	**CM5**	**NPA**	**AIM**	**Becke**	**MK**	**CHELPG**	**RESP**	**PEOE**
**C1**	C8	0.18	-0.26	0.01	0.02	0.08	0.06	0.36	0.18	0.33	0.30	0.31	0.06
**C2**	C6	-0.10	-0.20	-0.03	-0.12	0.00	-0.10	0.32	-0.02	-0.38	-0.28	-0.38	0.02
**H2**	H7	0.15	0.14	0.06	0.15	0.12	0.22	0.09	0.07	0.22	0.18	0.22	0.08
**N1**	N4	-0.23	0.19	-0.04	-0.11	-0.40	-0.49	-1.20	-0.13	-0.09	-0.17	-0.09	-0.31
**H1**	H5	0.23	0.19	0.16	0.27	0.35	0.41	0.44	0.20	0.29	0.30	0.29	0.15
**C3**	C2	0.07	-0.29	0.05	-0.02	0.15	0.16	0.97	0.18	-0.02	0.09	-0.02	0.08
**H3**	H3	0.15	0.13	0.06	0.15	0.13	0.20	0.09	0.07	0.15	0.11	0.15	0.10
**N2**	N1	-0.39	0.08	-0.18	-0.29	-0.37	-0.49	-1.14	-0.53	-0.35	-0.39	-0.34	-0.24
**C4**	C9	-0.27	-0.20	-0.05	-0.15	-0.14	-0.43	0.05	-0.17	-0.30	-0.18	-0.27	0.04
**H4A**	H10	0.14	0.17	0.05	0.12	0.11	0.25	0.06	0.12	0.12	0.08	0.12	0.04
**H4B**	H11	0.12	0.16	0.04	0.11	0.10	0.23	0.03	0.12	0.13	0.08	0.12	0.04
**C5**	C12	-0.11	-0.23	0.03	0.07	0.01	-0.15	0.22	-0.14	0.09	0.11	0.08	0.08
**H5**	H13	0.12	0.15	0.04	0.10	0.11	0.21	0.03	0.09	0.03	0.00	0.03	0.10
**N3**	N14	-0.26	0.15	0.00	-0.13	-0.59	-0.67	-1.03	-0.31	-0.12	-0.12	-0.09	0.24
**H3A**	H16	0.26	0.20	0.17	0.25	0.34	0.42	0.41	0.25	0.23	0.23	0.22	0.20
**H3B**	H17	0.29	0.19	0.13	0.22	0.33	0.46	0.50	0.30	0.23	0.23	0.23	0.20
**H3C**	H15	0.26	0.18	0.11	0.16	0.31	0.44	0.48	0.26	0.11	0.10	0.10	0.20
**C6**	C18	0.28	-0.66	0.13	-0.22	0.19	0.67	1.77	1.40	0.63	0.68	0.63	0.40
**O1**	O19	-0.42	0.00	-0.37	-0.28	-0.42	-0.68	-1.23	-0.97	-0.64	-0.66	-0.64	-0.24
**O2**	O20	-0.49	-0.07	-0.37	-0.30	-0.43	-0.75	-1.23	-0.98	-0.66	-0.69	-0.66	-0.24

Table S27 Histidine: ωB97XD/6-31g.

**Atom**	**AtomQM**	**Mulliken**	**Loewdin**	**Hirshfeld**	**ADCH**	**CM5**	**NPA**	**AIM**	**Becke**	**MK**	**CHELPG**	**RESP**	**PEOE**
**C1**	C8	0.27	-0.08	0.01	-0.04	0.08	0.05	0.44	0.23	0.34	0.30	0.32	0.06
**C2**	C6	-0.06	-0.13	-0.03	-0.20	-0.01	-0.10	0.39	0.03	-0.39	-0.29	-0.38	0.02
**H2**	H7	0.19	0.21	0.07	0.17	0.13	0.26	0.07	0.08	0.24	0.20	0.24	0.08
**N1**	N4	-0.36	-0.22	-0.04	-0.08	-0.40	-0.57	-1.35	-0.16	-0.13	-0.20	-0.13	-0.31
**H1**	H5	0.34	0.28	0.16	0.29	0.35	0.46	0.47	0.20	0.31	0.32	0.31	0.15
**C3**	C2	0.28	-0.02	0.05	0.24	0.15	0.17	1.15	-0.04	0.00	0.10	-0.01	0.08
**H3**	H3	0.18	0.21	0.07	-0.05	0.14	0.25	0.07	0.26	0.16	0.12	0.16	0.10
**N2**	N1	-0.35	-0.32	-0.18	-0.28	-0.38	-0.53	-1.31	-0.58	-0.37	-0.41	-0.36	-0.24
**C4**	C9	-0.87	-0.26	-0.05	-0.16	-0.14	-0.51	0.08	-0.19	-0.33	-0.20	-0.30	0.04
**H4A**	H10	0.22	0.22	0.06	0.13	0.12	0.30	0.05	0.13	0.14	0.10	0.13	0.04
**H4B**	H11	0.20	0.21	0.05	0.11	0.11	0.28	0.02	0.13	0.14	0.09	0.13	0.04
**C5**	C12	0.04	-0.13	0.03	0.07	0.01	-0.18	0.27	-0.10	0.06	0.08	0.05	0.08
**H5**	H13	0.19	0.20	0.04	0.11	0.11	0.27	0.02	0.10	0.05	0.01	0.05	0.10
**N3**	N14	-0.62	-0.26	0.00	-0.13	-0.59	-0.81	-1.18	-0.32	-0.13	-0.13	-0.10	0.24
**H3A**	H16	0.38	0.29	0.17	0.26	0.35	0.47	0.44	0.27	0.24	0.24	0.23	0.20
**H3B**	H17	0.41	0.31	0.14	0.23	0.33	0.50	0.54	0.31	0.25	0.25	0.24	0.20
**H3C**	H15	0.41	0.29	0.11	0.17	0.31	0.48	0.53	0.27	0.12	0.11	0.11	0.20
**C6**	C18	0.33	0.17	0.13	-0.20	0.20	0.72	1.86	1.26	0.71	0.76	0.71	0.40
**O1**	O19	-0.55	-0.45	-0.39	-0.30	-0.43	-0.71	-1.28	-0.94	-0.68	-0.70	-0.68	-0.24
**O2**	O20	-0.61	-0.52	-0.39	-0.34	-0.44	-0.78	-1.29	-0.95	-0.72	-0.74	-0.72	-0.24

Table S28 Histidine: ωB97XD/6-311g.

Atom	AtomQM	Mulliken	Loewdin	Hirshfeld	ADCH	CM5	NPA	AIM	Becke	MK	CHELPG	RESP	PEOE
**C1**	C8	0.57	-0.13	0.01	-0.05	0.08	0.07	0.40	0.22	0.33	0.29	0.31	0.06
**C2**	C6	-0.04	-0.06	-0.03	-0.21	-0.01	-0.08	0.34	0.03	-0.40	-0.30	-0.40	0.02
**H2**	H7	0.16	0.09	0.06	0.16	0.13	0.23	0.08	0.08	0.24	0.19	0.24	0.08
**N1**	N4	-0.23	0.04	-0.04	-0.07	-0.40	-0.53	-1.22	-0.15	-0.10	-0.18	-0.10	-0.31
**H1**	H5	0.36	0.13	0.16	0.29	0.35	0.42	0.43	0.20	0.30	0.31	0.30	0.15
**C3**	C2	0.19	-0.01	0.05	0.27	0.15	0.21	1.02	-0.02	-0.02	0.08	-0.02	0.08
**H3**	H3	0.15	0.09	0.07	-0.07	0.14	0.21	0.09	0.24	0.16	0.12	0.16	0.10
**N2**	N1	-0.26	-0.18	-0.18	-0.28	-0.38	-0.54	-1.19	-0.58	-0.35	-0.40	-0.35	-0.24
**C4**	C9	-1.36	-0.08	-0.05	-0.16	-0.14	-0.43	0.04	-0.18	-0.34	-0.20	-0.31	0.04
**H4A**	H10	0.23	0.11	0.06	0.13	0.12	0.25	0.07	0.13	0.14	0.10	0.14	0.04
**H4B**	H11	0.23	0.11	0.05	0.11	0.11	0.24	0.04	0.13	0.15	0.09	0.14	0.04
**C5**	C12	-0.13	-0.06	0.02	0.06	0.01	-0.11	0.24	-0.10	0.06	0.07	0.04	0.08
**H5**	H13	0.21	0.10	0.04	0.10	0.11	0.22	0.04	0.10	0.05	0.01	0.05	0.10
**N3**	N14	-0.34	0.19	0.00	-0.13	-0.59	-0.69	-1.06	-0.31	-0.13	-0.12	-0.10	0.24
**H3A**	H16	0.33	0.15	0.17	0.26	0.35	0.42	0.41	0.26	0.24	0.24	0.23	0.20
**H3B**	H17	0.34	0.15	0.14	0.23	0.33	0.46	0.50	0.31	0.25	0.25	0.24	0.20
**H3C**	H15	0.38	0.13	0.11	0.17	0.31	0.45	0.49	0.27	0.12	0.11	0.10	0.20
**C6**	C18	0.02	-0.13	0.13	-0.17	0.20	0.72	1.76	1.29	0.72	0.76	0.71	0.40
**O1**	O19	-0.39	-0.29	-0.38	-0.31	-0.43	-0.71	-1.23	-0.94	-0.68	-0.70	-0.68	-0.24
**O2**	O20	-0.43	-0.36	-0.38	-0.34	-0.44	-0.79	-1.23	-0.96	-0.72	-0.74	-0.72	-0.24

Table S29 Histidine: ωB97XD/def2tzvp.

Atom	AtomQM	Mulliken	Loewdin	Hirshfeld	ADCH	CM5	NPA	AIM	Becke	MK	CHELPG	RESP	PEOE
**C1**	C8	0.17	-0.26	0.01	-0.05	0.08	0.05	0.39	0.21	0.33	0.29	0.31	0.06
**C2**	C6	-0.12	-0.20	-0.03	-0.20	-0.01	-0.10	0.34	0.02	-0.39	-0.29	-0.39	0.02
**H2**	H7	0.17	0.14	0.06	0.16	0.13	0.23	0.09	0.07	0.23	0.19	0.23	0.08
**N1**	N4	-0.24	0.19	-0.04	-0.09	-0.40	-0.50	-1.25	-0.15	-0.10	-0.17	-0.10	-0.31
**H1**	H5	0.25	0.20	0.16	0.29	0.35	0.42	0.45	0.21	0.30	0.31	0.30	0.15
**C3**	C2	0.07	-0.29	0.05	0.25	0.15	0.17	1.02	-0.01	-0.02	0.09	-0.03	0.08
**H3**	H3	0.16	0.13	0.07	-0.05	0.14	0.21	0.10	0.22	0.15	0.12	0.15	0.10
**N2**	N1	-0.43	0.07	-0.18	-0.27	-0.38	-0.49	-1.20	-0.55	-0.35	-0.39	-0.34	-0.24
**C4**	C9	-0.26	-0.20	-0.05	-0.16	-0.14	-0.43	0.04	-0.19	-0.34	-0.21	-0.30	0.04
**H4A**	H10	0.15	0.17	0.06	0.13	0.12	0.26	0.07	0.13	0.14	0.09	0.13	0.04
**H4B**	H11	0.14	0.16	0.05	0.11	0.11	0.24	0.04	0.13	0.15	0.10	0.13	0.04
**C5**	C12	-0.12	-0.24	0.02	0.04	0.01	-0.15	0.22	-0.13	0.07	0.08	0.06	0.08
**H5**	H13	0.13	0.15	0.04	0.11	0.11	0.21	0.04	0.09	0.05	0.02	0.04	0.10
**N3**	N14	-0.27	0.15	0.00	-0.14	-0.60	-0.67	-1.08	-0.32	-0.14	-0.14	-0.10	0.24
**H3A**	H16	0.29	0.20	0.17	0.26	0.35	0.42	0.42	0.26	0.24	0.24	0.23	0.20
**H3B**	H17	0.30	0.20	0.14	0.23	0.33	0.46	0.51	0.31	0.24	0.24	0.24	0.20
**H3C**	H15	0.28	0.18	0.11	0.17	0.32	0.45	0.50	0.26	0.12	0.11	0.11	0.20
**C6**	C18	0.34	-0.67	0.13	-0.16	0.20	0.69	1.84	1.35	0.63	0.68	0.63	0.40
**O1**	O19	-0.46	0.00	-0.38	-0.31	-0.43	-0.69	-1.26	-0.96	-0.64	-0.66	-0.64	-0.24
**O2**	O20	-0.53	-0.07	-0.38	-0.33	-0.44	-0.77	-1.27	-0.96	-0.67	-0.69	-0.67	-0.24
